# Group-theoretical analysis of 1:3 *A*-site-ordered perovskite formation

**DOI:** 10.1107/S2053273318018338

**Published:** 2019-02-28

**Authors:** Mikhail V. Talanov

**Affiliations:** a Southern Federal University, Rostov-on-Don, Russian Federation

**Keywords:** quadruple 1:3 *A*-site-ordered perovskites, group-theoretical analysis, low-symmetry phases, full set of order parameters, tilts of octahedra, archetype structure

## Abstract

The quadruple perovskites *AA*′_3_ *B*_4_ *X*_12_ are characterized by an extremely wide variety of intriguing physical properties, which makes them attractive candidates for various applications. Using group-theoretical analysis, possible 1:3 *A*-site-ordered low-symmetry phases have been found. They can be formed from a parent  perovskite structure (archetype) as a result of real or hypothetical (virtual) phase transitions due to different structural mechanisms (orderings and displacements of atoms, tilts of octahedra). For each type of low-symmetry phase, the full set of order parameters (proper and improper order parameters), the calculated structure, including the space group, the primitive cell multiplication, splitting of the Wyckoff positions and the structural formula were determined. All ordered phases were classified according to the irreducible representations of the space group of the parent phase (archetype) and systematized according to the types of structural mechanisms responsible for their formation. Special attention is paid to the structural mechanisms of formation of the low-symmetry phase of the compounds known from experimental data, such as: CaCu_3_Ti_4_O_12_, CaCu_3_Ga_2_Sn_2_O_12_, CaMn_3_Mn_4_O_12_, Ce_1/2_Cu_3_Ti_4_O_12_, LaMn_3_Mn_4_O_12_, BiMn_3_Mn_4_O_12_ and others. For the first time, the phenomenon of variability in the choice of the proper order parameters, which allows one to obtain the same structure by different group-theoretical paths, is established. This phenomenon emphasizes the fundamental importance of considering the full set of order parameters in describing phase transitions. Possible transition paths from the archetype with space group  to all 1:3 *A*-site-ordered perovskites are illustrated using the Bärnighausen tree formalism. These results may be used to identify new phases and interpret experimental results, determine the structural mechanisms responsible for the formation of low-symmetry phases as well as to understand the structural genesis of the perovskite-like phases. The obtained non-model group-theoretical results in combination with crystal chemical data and first-principles calculations may be a starting point for the design of new functional materials with a perovskite structure.

## Abstract 2

A group-theoretical analysis of 1:3 *A*-site-ordered perovskite structures is reported.

## 1. Introduction   

*A*-site-ordered quadruple *AA*′_3_ *B*_4_ *X*_12_ perovskites occupy a special place among a large variety of functional materials (Mitchel, 2002 ▸; Tilley, 2016 ▸; King & Woodward, 2010 ▸; Aleksandrov & Beznosikov, 1997 ▸; Shimakawa, 2008 ▸; Yamada, 2017 ▸; Long, 2016 ▸; Vasil’ev & Volkova, 2007 ▸). This numerous family of materials is characterized by intriguing physical properties such as a giant dielectric constant (Subramanian *et al.*, 2000 ▸; Homes *et al.*, 2001 ▸), positive and negative magnetodielectricity (Imamura *et al.*, 2008 ▸), low- and high-field magnetoresistance (Chen *et al.*, 2014 ▸; Kida *et al.*, 2012 ▸), multiferroic properties (Wang *et al.*, 2015 ▸; Zhou *et al.*, 2017 ▸), large negative and positive thermal expansion (Long, Hayashi *et al.*, 2009 ▸; Zhang *et al.*, 2014 ▸; Long & Shimakawa, 2010 ▸) and heavy Fermion-like behavior (Kobayashi *et al.*, 2004 ▸).

1:3 *A*-site-ordered perovskites were discovered in 1967 (Deschanvres *et al.*, 1967 ▸). Interest in this class of materials increased sharply 33 years after the discovery in CaCu_3_Ti_4_O_12_ of a giant low-frequency dielectric constant (∊_0_ ≃ 10^5^), which is stable over a wide temperature range from 100 to 600 K (Subramanian *et al.*, 2000 ▸; Homes *et al.*, 2001 ▸). This discovery allowed CaCu_3_Ti_4_O_12_ to be considered as a possible material for devices, in particular in elements of static and dynamic random access memory. The giant dielectric constant in CaCu_3_Ti_4_O_12_ is usually associated with an extrinsic contribution, in particular with the internal barrier layer capacitance (Sinclair *et al.*, 2002 ▸; Cohen *et al.*, 2003 ▸; Tselev *et al.*, 2004 ▸; Adams *et al.*, 2006 ▸). This is due to the presence of a heterogeneous microstructure in the sample (semiconducting grains separated by insulating grain boundaries), which results in Maxwell–Wagner relaxation. Despite the extrinsic mechanism of the appearance of the giant dielectric constant in CaCu_3_Ti_4_O_12_, widely discussed in the literature, a number of works emphasize possible intrinsic contributions to the dielectric constant value (Liu *et al.*, 2005 ▸; Zhu *et al.*, 2007 ▸).

The discovery of the temperature-induced *A*–*B* intersite charge transfer effect, which is associated with large negative thermal expansion in LaCu_3_Fe_4_O_12_, aroused great interest (Long, Hayashi *et al.*, 2009 ▸). Later, charge transfer between the different transition-metal cations at the square-planar *A*′ and octahedral *B* sites was found in other 1:3 *A*-site-ordered perovskites: BiCu_3_Fe_4_O_12_ (Long & Shimakawa, 2010 ▸; Long, Saito *et al.*, 2009 ▸), LaCu_3_Cr_4_O_12_ and YCu_3_Cr_4_O_12_ (Zhang *et al.*, 2014 ▸). The latter two compounds showed positive thermal expansion-like volume changes at the intersite charge transfer transition. These materials can be used in such applications as elasticity-tuned sensors and switching devices (Long, Hayashi *et al.*, 2009 ▸). Note the site-selective electron doping effect, which is associated with a change in the valence in either the *A*′ or the *B* sites, depending on the choice of the type of atom in the *A* site (Zhang, Saito, Mizumaki *et al.*, 2013 ▸).

A wide variety of magnetic properties is also characteristic of 1:3 *A*-site-ordered perovskites which causes considerable scientific interest in these materials. For example, CaCu_3_(Ge,Sn)_4_O_12_ and (La,Dy)Cu_3_(Ge_3/4_Ga_1/4_)_4_O_12_ are ferromag­nets (Shimakawa, 2008 ▸; Shiraki *et al.*, 2007 ▸), CaMn_3_V_4_O_12_ and YMn_3_Al_4_O_12_ are antiferromagnets (Zhang, Saito, Chen *et al.*, 2013 ▸; Toyoda *et al.*, 2015 ▸), CaCu_3_Fe_4_O_12_, Ce_1/2_Cu_3_Ti_4_O_12_, BiCu_3_Mn_4_O_12_ and CaCu_3_Fe_2_Re_2_O_12_ are ferrimagnets (Chen *et al.*, 2014 ▸; Yamada *et al.*, 2008 ▸; Saito *et al.*, 2014 ▸; Takata *et al.*, 2007 ▸), (Na,Ca,Y)Cu_3_V_4_O_12_ are Pauli paramagnetics (Shimakawa, 2008 ▸; Zhang, Saito, Mizumaki *et al.*, 2013 ▸], and Ca(Mn_*x*_Cu_1−*x*_)_3_V_4_O_12_ and LuMn_3_V_4_O_12_ are spin-glass phases (Zhang, Saito, Chen *et al.*, 2013 ▸; Shimakawa *et al.*, 2014 ▸). The magnetic properties of these materials can be tuned both by changing the chemical composition (Shimakawa, 2008 ▸) and by cation (or vacancy) ordering (Saito *et al.*, 2014 ▸; Shimakawa *et al.*, 2014 ▸). An unusual geometric frustrated state in the Fe^3+^ spin sublattice was found in the *A*- and *B*-site-ordered quadruple perovskite CaCu_3_Fe_2_Sb_2_O_12_ (Chen *et al.*, 2014 ▸). The magnetic and transport properties of CaCu_3_Ru_4_O_12_ have attracted attention due to their heavy Fermion-like behavior (Kobayashi *et al.*, 2004 ▸). NaMn_3_Mn_4_O_12_ shows a sequence of phase transitions associated with the spin, charge and orbital orderings (Prodi *et al.*, 2004 ▸). Recently, a strong spin-driven magnetoelectric effect was observed in a perovskite LaMn_3_Cr_4_O_12_ with a centrosymmetric cubic structure (Wang *et al.*, 2015 ▸). It is assumed that ferroelectricity in this compound is of an electronic nature and is formed due to magnetic ordering in two magnetic sublattices.

Thanks to all these physical properties, 1:3 *A*-site-ordered quadruple *AA*′_3_ *B*_4_ *X*_12_ perovskites are still of great interest. The diverse and unique physical properties of 1:3 *A*-site-ordered perovskites are mainly due to the ordered structure of these crystals. We have previously carried out a study of cation- and anion-ordered low-symmetry modifications of the parent 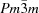 perovskite structure (Talanov, Shirokov *et al.*, 2014 ▸; Talanov, Talanov *et al.*, 2014 ▸; Talanov *et al.*, 2016 ▸). In this article, we emphasize the systematic study of perovskite-like structures with an order of 1:3 in the *A* sublattice which are formed as a result of real or hypothetical (virtual) structural phase transitions from the parent phase through various structural mechanisms.

Currently, there are two main theoretical approaches to the calculation of possible structures of the phases. The first approach is based on the *International Tables for Crystallography* (2010 ▸). We will call this the ITC approach for short. The ITC approach is described in detail by Bärnighausen (1975 ▸, 1980 ▸), Müller (2004 ▸, 2013 ▸). The second approach is based on group-theoretical methods of the Landau theory of phase transitions (Landau, 1937 ▸). It is based on the theory of irreducible representations (irreps) of the space symmetry groups. We will call it the ‘representation’ approach, or the R-approach. Here we describe briefly each of these approaches.

The ITC approach accumulates crystallographic knowledge gained over more than 100 years – since the discovery of space symmetry groups by Fedorov (1891 ▸) and Schoenflies (1891 ▸). The visual representation of symmetry relations between different structures/phases is given in the form of a hierarchical ‘family tree’ or Bärnighausen tree. The special Bärnighausen formalism is proposed, including information about the paths of transition from the initial high-symmetry structures/phases (aristotype) with space group *G* to ‘daughter’ low-symmetry structures/phases (hettotypes) with space group *H* indicating the kind of maximum subgroup and index of the symmetry reduction. According to the Hermann theorem (Hermann, 1928 ▸) there are two kinds of maximal subgroups: isotranslational (now called ‘translation­en­gleiche’, *i.e.* subgroups *H* have the same translation lattice as *G*) and isoclass (their name is ‘klassengleiche’, *i.e.* sub­groups *H* having a different translation lattice, but belonging to the same crystal class as *G*). In the Bärnighausen formalism these subgroups are denoted by ‘*tn*’ and ‘*kn*’, respectively, where *n* is an index of symmetry reduction (Müller, 2013 ▸). From the kinds of subgroups it is possible to deduce what and how many kinds of domains can result from a phase transition or topotactic reaction (Lotgering, 1959 ▸; Giovanoli & Leuenberger, 1969 ▸) involving a symmetry reduction. In addition, changes in the basis vectors, the origin shift and the splitting of Wyckoff positions are indicated. Numerous examples of the application of the ITC approach are given by Müller (2013 ▸) and Bärnighausen (1980 ▸). This approach is a useful tool for solving the structure of new crystals as well as for the structural design of novel materials.

The R-approach has been widely used in the investigation of low-symmetry structures of crystals and structural mechanisms of phase transitions ever since the classical works of Landau and Lifshitz (Landau, 1937 ▸; Lifshitz, 1941 ▸; Landau & Lifshitz, 1976 ▸). It consists of two stages. The first stage includes finding all possible low-symmetry phases, corresponding order parameters (OPs), basic vectors of primitive cells and changing of primitive cell volume (*V*/*V*_0_) for the irrep of the space group in the parent phase. At the second stage, the structures of the low-symmetry phases are determined. This problem was examined by Sakhnenko *et al.* (1986 ▸) and a general method for its solution was suggested there. The structure of the low-symmetry phase is connected with the so-called ‘complete condensate of OPs’ [the full set of the proper (primary) and the improper (secondary) OPs] as well as with the basis functions of irreps.

The group-theoretical analysis is quite a cumbersome procedure requiring the use of complex computer programs especially if OPs are multicomponent. There are some software applications for solution of the group-theoretical tasks: *CONDENSATE* and *BASIS* (Chechin, 1989 ▸), *ISOTROPY* (Howard & Stokes, 2005 ▸; Stokes & Hatch, 2002 ▸), the Bilbao Crystallographic Server (Aroyo *et al.*, 2006 ▸; Perez-Mato *et al.*, 2010 ▸) and others.

Visualization of the crystallographic analysis results of new structures is an attractive part of the ITC approach. This is used in the routine practice of solving structures. The R-approach is less visual, but it allows one to describe and predict not only atom but also spin and orbital structures, as well as predict important physical effects, revealing the physical nature of proper and improper OPs. This approach is naturally linked with phenomenological thermodynamics, quantum mechanics, optics, magnetism, orbital physics and other fields of science.

The use of only one of the above approaches (ITC or R) considerably reduces the possibilities of applying the obtained results to solving various theoretical and practical problems of crystallography and other sciences associated with it. These two approaches complement each other. In our study of 1:3 *A*-site-ordered perovskite-like structures, an emphasis will be placed on the R-approach. But in the final part of the article, the Bärnighausen formalism will be used to visualize the genesis of 1:3 *A*-ordered perovskite-like structures and to establish the relationship between the ITC and R-approaches.

C. J. Howard and H. T. Stokes were the first to show that the 1:3 *A*-site ordering is associated with an OP, transforming by the irrep **k**_11_τ_1_(*M*_1_^+^) (Howard & Stokes, 2005 ▸). This study relies on the above work and supplements it. It will be shown that 1:3 *A*-site-ordered low-symmetry phases can be formed in different ways. A group-theoretical analysis of the structural mechanisms of formation of 1:3 *A*-site-ordered low-symmetry phases, as well as a group-theoretical map of their generation paths, are reported.

In this work, the possible structures of *A*-site-ordered quadruple *AA*′_3_ *B*_4_ *X*_12_ perovskites have been found based on powerful group-theoretical methods of Landau phase transition theory (Howard & Stokes, 2005 ▸; Howard *et al.*, 2003 ▸; Aroyo *et al.*, 2006 ▸; Perez-Mato *et al.*, 2010 ▸; Toledano & Toledano, 1987 ▸; Toledano & Dmitriev, 1996 ▸; Birman, 1978 ▸; Vinberg *et al.*, 1974 ▸; Aleksandrov & Bartolomé, 2001 ▸; Bock & Müller, 2002 ▸; Balachandran & Rondinelli, 2013 ▸; Stokes & Hatch, 1988 ▸; Chechin, 1989 ▸; Talanov *et al.*, 2015 ▸, 2018 ▸; Talanov & Shirokov, 2014 ▸; Talanov, 2007 ▸, 2018 ▸; Stokes & Campbell, 2017 ▸). The *ISOTROPY* software suite was used for the calculations (Howard & Stokes, 1998 ▸, 2004 ▸, 2005 ▸; Campbell *et al.*, 2006 ▸). In particular, the *ISOSUBGROUP* program was used to obtain the list of low-symmetry phases induced by various OPs, as well as to determine the improper OPs. For a detailed study of the low-symmetry phase structure (splitting of the Wyckoff positions, obtaining different domains of the same phase *etc*.), the *ISOTROPY* program and ITC (2010 ▸) were used.

Thus, the aim of this study is a group-theoretical analysis of possible pathway formation and structural genesis of 1:3 *A*-site-ordered perovskites using the ITC and R-approaches.

## 2. Paths of 1:3 *A*-site-ordered perovskite formation   

The space group of the *ABX*_3_ cubic perovskite structure is 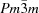. In this structure, *A* cations occupy Wyckoff position 1*a* with cubo-octahedral coordination and with local symmetry 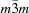, octahedral *B* cations occupy Wyckoff position 1*b* with local symmetry 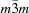 and *X* anions occupy Wyckoff position 3*c* with local symmetry 4/*mm.m*. Perovskites are referred to as anion-octahedral-type structures, *i.e. BX*_6_ octahedra act as building blocks which are connected together by common vertices in three directions and are separated by *A* cations located in cubo-octahedral voids. Note that 1*a* and 1*b* positions in the perovskite structure are symmetrically equivalent. They are connected by an external automorphism, *i.e.* origin shift (½ ½ ½) of the unit cell. This means that the origin can be chosen both at *A*- (like in this work) and *B*-cation sites. The fact that the irreps responsible for these two descriptions are different must be taken into account.

The idealized perovskite structure is the archetype (this word originates from the ancient Greek one ‘αρρχ∊τυπου’ which means ‘prototype’) and/or aristotype (from the ancient Greek word ‘αριστοξ’, ‘highest’) for low-symmetry modifications (Megaw, 1957 ▸, 1973 ▸; Aleksandrov *et al.*, 1981 ▸; Talanov *et al.*, 2016 ▸).^1^ Atom displacements (including ordered tilts or rotations of the *BX*_6_ octahedra) and orderings, as well as their combinations, result in lower-symmetry superstructures.

In the Landau theory, the low-symmetry structure formation is described by proper and improper OPs, which are transformed according to the irrep of the space symmetry group of the high-symmetry parent phase. Irreps (according to which a proper OP is transformed) determine the symmetry and structural motif of low-symmetry phases near the transition point. However, when a low-symmetry structure is far from the temperature of the phase transition (*T*_c_), the contribution of improper OPs to atom displacements (and/or orderings) can become essential (Dimmock, 1963 ▸; Bruce & Cowley, 1981 ▸; Sakhnenko *et al.*, 1986 ▸; Molokeev & Misyul’, 2012 ▸). The appearance of these contributions is connected with nonlinear interactions between different degrees of freedom in a crystal. There are two main notation schemes of irreps: by Kovalev and by Miller-Love.^2^ In the first case, the designation is **k**_*n*_τ_*m*_, where *n* is the number of the wavevector **k** and *m* is the number of the corresponding irrep. In the second case, the wavevectors are denoted by different capital letters (Γ, *M*, *X* …), and numbers of irreps are designated as subscript numbers and superscript signs ‘+’ and ‘−’. We will use the designations for irreps according to both said schemes. In addition, the OP direction in the OP space is necessary to indicate its unambiguous identification. The OPs transformed by irreps with different **k** values will be denoted by various Greek letters (η, φ, σ, Ψ). In this article, we will consider only those irreps that satisfy the Lifshitz criterion (Landau & Lifshitz, 1976 ▸), *i.e.* they induce commensurate phases.

The full set of proper and improper OPs fully determines the structure and all possible symmetry-dependent properties of the crystal (Sakhnenko *et al.*, 1986 ▸). The analysis of the full set of the OPs allows conclusions to be drawn about the nature of the crystal properties. The proper OPs that are responsible for the manifestation of ferroelectric properties, orderings and atom displacements, tilts of anion octahedra and Jahn–Teller distortions in perovskites are well known (Howard & Stokes, 1998 ▸, 2004 ▸, 2005 ▸; Howard *et al.*, 2003 ▸; Carpenter & Howard, 2009 ▸; Howard & Carpenter, 2010 ▸; Senn & Bristowe, 2018 ▸; Talanov, Shirokov *et al.*, 2014 ▸; Talanov, Talanov *et al.*, 2014 ▸).

In Table 1 ▸, irreps according to which OPs are transformed inducing atom displacements^3^ (polar and non-polar), atom orderings^4^ and tilts of anion octahedra (or tilt-like distortions) in perovskites are summarized.

**Figure 1:**
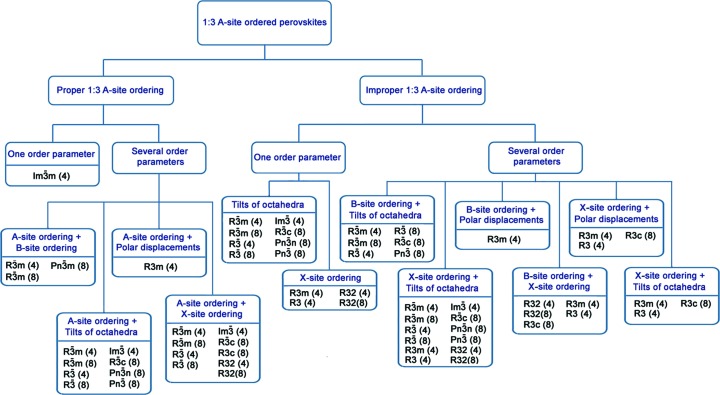
A classification of the OP combinations of 1:3 *A*-site-ordered perovskites that are considered in this work. The change in the primitive cell volume of the low-symmetry phase relative to the parent perovskite structure with the 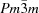 space group is indicated in brackets after the designation of the space group.

Let us return to the analysis of the ways of 1:3 *A*-ordered phase formation (Fig. 1 ▸). Irrep **k**_11_τ_1_(*M*_1_^+^) may in different ways enter into a full set of OPs, describing different mechanisms of 1:3 *A*-site-ordered phase formation. These OPs can be either proper or improper OPs. In addition, they can form combinations with other OPs. Thus, there are two types of mechanism of 1:3 *A*-site-ordered perovskite formation associated with proper and improper atom orderings. For both mechanisms, further analysis is connected with the number of proper OPs. For mechanisms of the first type, proper ordering in perovskite *A* sublattices and its combinations with other OPs are considered. For mechanisms of the second type, tilts of anion octahedra, atom ordering in the *X* sublattice, as well as combinations of these mechanisms with ordering in the *B* sublattice and atom displacements, are considered.

To enumerate all the low-symmetry phases formed as a result of atom displacements, it is necessary to use the OPs that are transformed by the irreps taken from the composition of the mechanical (atom-displacement) representation of the perovskite structure. This representation includes 20 different irreps (Table 1 ▸), combinations of which induce more than 1200 low-symmetry phases. Because of the cumbersome nature of the complete list of all low-symmetry phases formed as a result of atom displacements, the study is limited to irrep **k**_12_τ_10_(Γ_4_^−^) only. This irrep is associated with the polar displacements of atoms. The research focuses on phases with polar displacements, because these phases possess ferroelectric properties.

An analysis of Table 1 ▸ showed that there is no irrep that is included simultaneously in the permutation representations on both the *A* and *B* sites in the perovskite structure. This means that there cannot exist a single OP that describes the formation of a perovskite-like structure with simultaneous *A*- and *B*-site ordering. For this reason, in Fig. 1 ▸ and in the present work, *B*-site ordering is considered only in combinations with other OPs, but not as ‘one order parameter’.

Many phases with the same structures can be obtained by different paths (Fig. 1 ▸). For example, the structure of the rhombohedral phase with space group 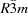 and *V*/*V*_0_ = 4 can be induced by the tilts of octahedra or by the combination of *A*-site ordering and *X*-site ordering or by the combination of *A*-site ordering and *B*-site ordering *etc*. This complicates the analysis of similar phases in Table 2 ▸, where the proper and improper OPs, the primitive cell translations and the changes in their volumes, as well as structural formulas are given. Therefore, in what follows, only some (possibly minimal) sets of proper OPs from the full set of OPs will be given for these phases.

## 3. Structural mechanisms of 1:3 *A*-site-ordered perovskite formation   

### 3.1. Proper ordering in the perovskite *A* sublattice   

#### 3.1.1. Case of one OP   

There are no experimentally observed phase transitions from the parent phase with the cubic perovskite structure (archetype) to 1:3 *A*-site-ordered perovskite phases. Nevertheless, within the framework of the archetype concept, the genesis of various ordered phases from an archetype and the structural mechanism of their formation can be determined. Of course, such a structural mechanism is conditional. However, it plays a crucial role as it allows one to understand possible types of atom displacements and permutations that can cause the formation of the 1:3 *A*-site-ordered perovskite phases from the archetype structure. Thus, by using the concept of structural mechanisms, the similarity, hierarchy and genesis of the studied crystal structures can be shown.

To list all possible low-symmetry phases with ordering in the *A* sublattice, obtained from the archetype with the space group 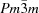, we consider the corresponding permutation representation on the 1*a* Wyckoff position of the perovskite structure (Table 1 ▸). It contains the following set of irreps:
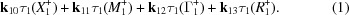



The irreps describing the ordering of atoms in the 1*a* (and 1*b*) Wyckoff positions of the perovskite structure for the *X*, *M* and Γ points of the Brillouin zone are 3D, and for the *R* point the irrep is 1D. The irrep **k**_12_τ_1_(Γ_1_^+^) is the unit irrep and does not lead to the atom ordering.

It is known that four binary low-symmetry *A*-site-ordered perovskite phases with space groups 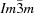 (type of order is 1:3), 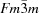 (type of order is 1:1) and two different phases with space groups *P*4/*mmm* (type of order is 1:1) are possible (Talanov, Talanov *et al.*, 2014 ▸). However, only one of these phases with the 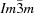 space group is characterized by 1:3 *A*-site ordering (Table 2 ▸). The three-component OP is transformed by the irrep **k**_11_τ_1_(*M*_1_^+^) entering into the permutation representation (1). This irrep also enters into the mechanical representation on the 3*c* Wyckoff position that leads to anion displacements in the ordered phase formation (Table 1 ▸). In this case, the structural formula of the ordered phase must be *A* ^2*a*^_1/4_ *A* ^6*b*^_3/4_ *B* ^8*c*^ *X* ^24*h*^_3_.

**Figure 2:**
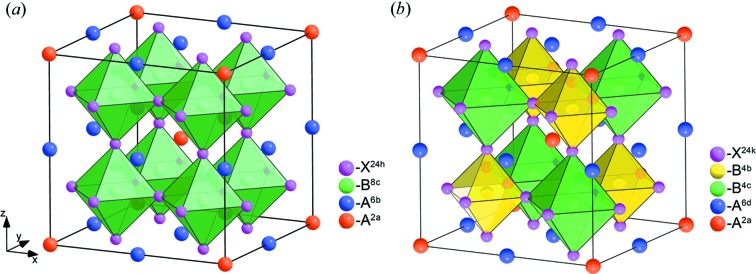
Calculated low-symmetry 1:3 *A*-site-ordered perovskite structures with space groups (*a*) 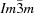 and (*b*) 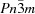.

The given structure is an aristotype for a large number of derivatives, which can be obtained from this structure by *B*-site ordering, tilts of anion octahedra and other mechanisms. In this structure cation chains *A* ^6*b*^–*A* ^6*b*^–*A* ^6*b*^ and *A* ^6*b*^–*A* ^2*a*^–*A* ^6*b*^ are oriented in the directions [100], [010] and [001]. In the [*B* ^8*c*^ *X* ^24*h*^_6_] octahedra, the *X* ^24*h*^ atoms are located at the same distance from the central atom (Fig. 2 ▸ *a*). The octahedra are linked to each other only by the vertices. There are not many substances with such a structure. This is explained by the fact that *BX*_6_ octahedral distortions (in particular, tilts) are strongly dependent on the *A*-site cation size (Goldschmidt, 1926 ▸; Megaw & Darlington, 1975 ▸; Thomas & Beitollahi, 1994 ▸; Woodward, 1997*b* ▸; Aso *et al.*, 2014 ▸). As a result of distortions in the structure that arise due to dimensional mismatch of ions, additional OPs, in particular, the tilts of anion octahedra, will appear. Rare examples of the realization of this crystal structure are bismuth-containing superconducting oxides, (Na_0.25_K_0.45_)(Ba_1.00_)_3_(Bi_1.00_)_4_O_12_, (K_1.00_)(Ba_1.00_)_3_(Bi_0.89_Na_0.11_)_4_O_12_ and Ba_1−*x*_K_*x*_Bi_1−*y*_Na_*y*_O_3_, obtained by hydro­thermal synthesis (Rubel *et al.*, 2014 ▸, 2016 ▸; Zhang *et al.*, 2011*b* ▸).

#### 3.1.2. Several OPs   

(i) *Combined proper atom ordering in *A* and *B* sublattices.* The appearance of cation order in the *A* sublattice can be associated with cation ordering in the *B* sublattice. In this case, the transition to a low-symmetry structure is described by two independent OPs, which are transformed by the corresponding irreps entered into the permutation representation on the 1*a* and 1*b* Wyckoff positions of the perovskite structure. We obtained various low-symmetry perovskite phases with simultaneous ordering in the *A* and *B* sublattices (Talanov, Shirokov *et al.*, 2014 ▸). We showed that there exist 121 phases with simultaneous cation ordering in the *A* and *B* sublattices, and only three structures with 1:3 order in the *A* sublattice: two 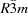 rhombohedral phases and one 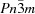 cubic phase (Table 2 ▸).

We consider in detail the structural mechanisms of 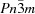 phase formation. This phase is an aristotype of a large family of quadruple perovskites which are sometimes referred to as 1322 perovskites (Senn *et al.*, 2014 ▸).^5^ This structure is generated by two proper OPs that are transformed by 4D reducible representation, formed as a direct sum of irreps **k**_11_τ_1_(*M*_1_^+^) and **k**_13_τ_4_(*R*_2_^−^). Irrep **k**_11_τ_1_(*M*_1_^+^) enters into permutation representation on the 1*a* Wyckoff position and also into mechanical representation on the 3*c* Wyckoff position of the perovskite structure. Irrep **k**_13_τ_4_(*R*_2_^−^) enters into permutation representation on the 1*b* Wyckoff position and also into mechanical representation on the 3*c* Wyckoff position. This means that low-symmetry phase formation is accompanied by simultaneous *A*-cation and *B*-cation ordering and also by anion displacements. According to the group-theoretical calculations the structural formula of the 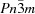 ordered perov­skite is as follows: *A* ^2*a*^_1/4_ *A* ^6*d*^_3/4_ *B* ^4*b*^_1/2_ *B* ^4*c*^_1/2_ *X* ^24*k*^_3_. The structure of this phase is shown in Fig. 2 ▸(*b*). In this structure, the distorted octahedra with the *B* ^4*c*^ and *B* ^4*b*^ central atoms are located in a checkerboard order. *A* ^2*a*^ and *A* ^6*d*^ cations in a ratio of 1:3 are ordered in voids formed by octahedra. The structure is characterized by an eightfold increase in the volume of the primitive cell. This theoretically predicted structure was found in loparite (Na_0.59_Ce_0.41_)_3_(Na_0.13_Ca_0.47_Ce_0.40_)Ti_2_(Ti_0.73_Nb_0.27_)_2_O_12_ (Zubkova *et al.*, 2000 ▸). The distribution of atoms on Wyckoff positions in the structure of this mineral exactly corresponds to the theoretical result obtained: *A* ^2*a*^(Na_0.13_Ca_0.47_ M*)_3_ *A* ^6*d*^ (Na_0.59_Ce_0.41_)_3_ *B* ^4*c*^(Ti_0.73_Nb_0.27_)_2_ *B* ^4*b*^(Ti)_1/2_ *X* ^24*k*^(O)_12_, where M* = Ce_0.19_La_0.08_Nd_0.04_Th_0.01_Sr_0.05_Pr_0.01_Eu_0.01_Sm_0.01_.

(ii) *Combined proper atom ordering in the *A* sublattice and polar displacements.* The combined OP, which transforms according to the direct sum of the irreps **k**_11_τ_1_(*M*_1_^+^) and **k**_12_τ_10_(Γ_4_^−^), generates only one 1:3 *A*-site-ordered phase with the space group *R*3*m* (more details in Section 3.2.2). The former irrep is responsible for the proper 1:3 *A*-site ordering, while the latter is responsible for the polar displacements. The structure of this phase is characterized by simultaneous atom ordering in all sublattices: 1:3 type of order in the *A* and *B* sublattices and 1:1:2 order type in the *X* sublattice. The ordering in the *B* and *X* sublattices is related to the improper OPs transformed by irrep **k**_11_τ_7_(*M*_4_^+^) and by the direct sum of irreps **k**_10_τ_4_(*X*_3_^−^), **k**_11_τ_7_(*M*_4_^+^), **k**_11_τ_10_(*M*_5_^−^), respectively. Anion octahedra in the structure of this phase are distorted (non-Glazer tilts) due to the contribution of the OP (φ −φ φ −φ φ −φ) transformed by irrep **k**_11_τ_9_(*M*_5_^+^). The polarization vector in this phase is directed along the solid diagonal [111]. This phase is similar to the structure generated by the OP associated with the ordering in the *X* sublattice, but it is an improper ferroelectric, which is discussed in the next section.

### 3.2. Improper ordering in the *A* sublattice   

#### 3.2.1. One OP   

(i) *Ordering of atoms in the *X* sublattice.* Atom ordering in the *A* sublattice can be a secondary effect of atom ordering in other perovskite sublattices. We have shown previously that the ordering in the *B* sublattice of the perovskite structure, described by a single OP, cannot lead to simultaneous ordering in the *A* sublattice (Talanov, Talanov *et al.*, 2014 ▸). Yet, the proposed ordering mechanism can be realized with anion ordering in the *X* sublattice (Talanov *et al.*, 2016 ▸). Of all the phases that are formed as a result of the anion ordering (induced by only one OP without the contribution of additional structural mechanisms), only three phases show 1:3 *A*-site ordering. These are rhombohedral phases with the space groups *R*3*m*, *R*3 and *R*32.

These phases are generated by one 6D irrep **k**_11_τ_10_(*M*_5_^−^), but characterized by different directions in OP space: (φ_1_ φ_2_ φ_1_ φ_2_ φ_1_ φ_2_), (φ φ φ φ φ φ) and (φ −φ φ −φ φ −φ). These phases have much in common:

(*a*) They are characterized by 1:3 *B*-site ordering, which is generated by improper irrep **k**_11_τ_7_(*M*_4_^+^); this irrep enters into the permutation representations on the 1*b* and 3*c* Wyckoff positions of the perovskite structure [as well as in the mechanical representation on the 3*c* Wyckoff position (Table 1 ▸)].

(*b*) They are characterized by quadruplications of the primitive volume cells relative to the parent perovskite structure.

(*c*) In the structures of these phases, tilt-like distortions generated by an improper OP transformed by irrep **k**_11_τ_9_(*M*_5_^+^) are possible. In the case of the *R*3 phase the full set of the OPs also includes irreps **k**_11_τ_5_(*M*_2_^+^) and **k**_12_τ_9_(Γ_4_^+^) (Table 1 ▸).

(*d*) Irrep **k**_11_τ_10_(*M*_5_^−^) also enters into mechanical representations on the 1*a* and 1*b* Wyckoff positions that describe the displacement of the *A* and *B* cations.

In addition, it is important to note that phases with *R*3*m* and *R*3 space groups are improper ferroelectrics (the structures of similar phases, which are proper ferroelectrics induced by the same full set of the OPs, are described in Section 3.1.2). All three phases are improper ferroelastics.

Besides, another phase with the *R*32 space group and *V*/*V*_0_ = 8 can be induced by two OPs transformed by the direct sum of irreps **k**_11_τ_10_(*M*_5_^−^) and **k**_10_τ_4_(*X*_3_^−^) (phase 4 of Table 2 ▸). The last irrep enters into the permutation representations on the 1*b* Wyckoff position. The structure of this phase can also be attributed to the structures formed as a result of combined *X*- and *B*-site ordering (Fig. 1 ▸).

(ii) *Tilts of anion octahedra.* The tilts of anion octahedra along with the atom displacements or orderings can also be OPs. Note that the tilts of anion octahedra are the most common type of distortions in perovskites (Megaw, 1957 ▸). Glazer and Alexandrov obtained 23 systems of possible tilts of anion octahedra in perovskites and proposed designations for their description (Glazer, 1972 ▸, 1975 ▸; Aleksandrov, 1976 ▸). Based on the results of the group-theoretical analysis, it was found that the OPs associated with tilts of anion octahedra are transformed by two irreps: **k**_11_τ_5_(*M*_2_^+^), in-phase rotation, and **k**_13_τ_8_(*R*_5_^−^), out-of-phase rotation. These OPs, as well as their combinations, initiate the formation of 14 types of structures with Glazer tilts of octahedra (Howard & Stokes, 1998 ▸; Campbell *et al.*, 2018 ▸).

In many studies, the superposition of the tilts of anion octahedra and other mechanisms of lattice distortions – atom displacements (Aleksandrov & Bartolomé, 2001 ▸; Aleksandrov, 1978 ▸; Stokes *et al.*, 2002 ▸; Torgashev *et al.*, 2005 ▸), cation orderings (Howard *et al.*, 2003 ▸, Howard & Stokes, 2004 ▸; Woodward, 1997*a* ▸), Jahn–Teller distortions (Balachandran & Rondinelli, 2013 ▸; Carpenter & Howard, 2009 ▸; Howard & Carpenter, 2010 ▸) – are considered. In the above works only ‘simple’ tilts of anion octahedra in-phase or out-of-phase were studied. However, as shown by Shirokov & Torgashev (2004 ▸), the tilts of anion octahedra in the perovskite structure can be induced not by two, but by six OPs, transforming according to the following irreps:
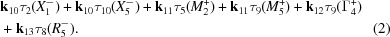



The absolute majority of perovskite-like structures with tilts of anion octahedra induced by one of the two OPs [**k**_11_τ_5_(*M*_2_^+^) and **k**_13_τ_8_(*R*_5_^−^)] or a combination of them can be described by the Glazer tilt system. As noted by Howard & Stokes (2005 ▸), distortions of structures generated by the 6D irreps **k**_10_τ_10_(*X*_5_^−^) and **k**_11_τ_9_(*M*_5_^+^) are not tilts of octahedra in Glazer approximations and, hence, cannot be described by the Glazer notation system. However, unconventional non-Glazer tilts are observed in hybrid organic–inorganic perovskites where additional degrees of freedom become possible (Duyker *et al.*, 2016 ▸; Wu *et al.*, 2018 ▸; Boström *et al.*, 2018 ▸; Wei *et al.*, 2018 ▸). That is why consideration of structures with non-Glazer tilts can be useful for describing possible types of distortions in a wide variety of compounds; therefore they are included in this section as ‘tilt-like distortions’.

In order to solve the problem of enumerating all possible low-symmetry phases obtained from the parent phase of 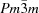 by the tilts of anion octahedra, we consider a four-component (24D) OP. This is transformed by the direct sum of the above irreps. This OP can generate the formation of 156 low-symmetry phases. However, only eight phases are characterized by 1:3 *A*-site ordering (Fig. 1 ▸), none of the phases being improper ferroelectrics.

The formation of one phase (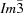) induced by the OP, transforming according to the irrep **k**_11_τ_5_(*M*_2_^+^), is accompanied by in-phase tilts of octahedra (*a* ^+^ *a* ^+^ *a* ^+^ Glazer tilt system) (Table 2 ▸). A phase with the 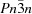 space group is induced by the OP, transforming according to the irrep **k**_10_τ_2_(*X*_1_^−^), which, in its turn, is associated with unconventional tilts of octahedra (Boström *et al.*, 2018 ▸).

Each of the remaining six phases can be obtained as a result of a combination of different OPs: three phases induced by the OPs, transforming according to irrep **k**_10_τ_10_(*X*_5_^−^), and two phases induced by the OPs, transforming according to irrep **k**_11_τ_9_(*M*_5_^+^). The former are formed with an eightfold increase in primitive cell volume, and the latter with a fourfold increase. In these structures, there is an ordering in the *B* sublattice induced by improper OPs that are transformed by irreps **k**_10_τ_4_(*X*_3_^−^), **k**_11_τ_7_(*M*_4_^+^), **k**_13_τ_4_(*R*_2_^−^), which enter into the permutation representation of perovskite structure on the 1*b* Wyckoff positions (Talanov *et al.*, 2016 ▸). The 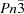 phase can be induced by a combination of several OPs only (see the next section).

**Figure 3:**
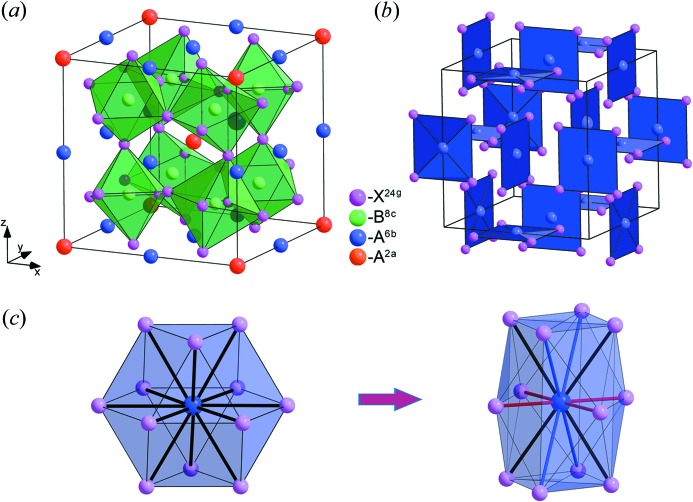
(*a*) Low-symmetry 1:3 *A*-site-ordered perovskite structures with space group 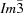. (*b*) Square-planar coordination of *A* ^6^ *^b^* cations. (*c*) The nearest environment of *A* ^6^ *^b^* cations in 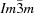 (left) and 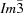 (right) structures. Short *A* ^6^ *^b^*—*X* ^24^ *^h^* ^/^ *^g^* bonds are highlighted in red, medium in black, long in blue.

We consider the 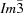 phase in more detail (phase 5 of Table 2 ▸, Fig. 3 ▸ *a*) because there are a large number of experimentally detected examples of such 1:3 *A*-site-ordered perovskites, in particular a family of CaCu_3_Ti_4_O_12_ materials (Subramanian *et al.*, 2000 ▸; Belik, 2018 ▸). The structure of this phase is generated by a single OP (φ φ φ), which is transformed by an irrep **k**_11_τ_5_(*M*_2_^+^). Two improper OPs (φ φ φ) and σ, transforming according to irreps **k**_11_τ_1_(*M*_1_^+^) and **k**_12_τ_3_(Γ_2_^+^), respectively, are also included in the full set of OPs.

As a result of the in-phase tilts of anion octahedra with large inclination amplitude [in CaCu_3_Ti_4_O_12_ the *B*—*X*—*B* bond angle is approximately equal to 140° (Bochu *et al.*, 1979 ▸)], a square-planar environment of *A* ^6*b*^ cations arises (Fig. 3 ▸ *b*). Thus, 12 identical distances *A*—*X* (in the case of undistorted perovskite) are divided into four short, four medium and four long ones (Fig. 3 ▸ *c*). This local configuration is stabilized by the Jahn–Teller effect in cases when the *A* ^6*b*^ positions are occupied by Cu^2+^ or Mn^3+^ ions. Rare examples of 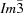 structure formation are the compounds with a substitution (total or partial) of Jahn–Teller ions on the non-Jahn–Teller ones: Fe^2+^ (Leinenweber *et al.*, 1995 ▸), Li^+^ (Mouron & Choisnet, 1987 ▸), Ti^4+^ (Li *et al.*, 2004 ▸; Avdeev & Nalbandyan, 2006 ▸), Co^2+^ (Ovsyannikov, Zainulin *et al.*, 2013 ▸), Pd^2+^ (Shiro *et al.*, 2013 ▸) and Pb^4+^ (Sakai *et al.*, 2017 ▸). In this case, a slight displacement of the *A* ^6*b*^ atom in the direction perpendicular to the plane of square-planar oxygen coordination (Leinenweber *et al.*, 1995 ▸; Ovsyannikov, Zainulin *et al.*, 2013 ▸; Sakai *et al.*, 2017 ▸) or splitting of *A* ^6*b*^ positions (Tohyama *et al.*, 2013 ▸; Shimura *et al.*, 2016 ▸) is possible.

Relatively large (Ca, Sr, Na, Pb, Bi) cations occupy the cubo-octahedral 12-coordinated *A* ^2*a*^ positions. However, there are cases where smaller ions of transition metals Mn^2+^ (Akizuki *et al.*, 2013 ▸) and Cu^2+^ (Akizuki *et al.*, 2015 ▸) occupy these positions. In the CuCu_3_V_4_O_12_ compound, there are significant temperature vibrations of Cu^2+^ ions in oversized cubo-octahedral cages, which are interpreted as a rattling effect (Jeitschko & Braun, 1977 ▸). It is interesting to note the structural similarity of 1:3 *A*-site-ordered perovskites with the 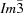 space group to the widely studied class of cage compounds filled skutterudites *A* ^2*a*^ *B*_4_^8*c*^ *X*_12_^24*g*^ (*A* = rare-earth element, *B* = transition metal, *X* = pnicogen) with the same space group (Sales *et al.*, 1996 ▸; Aleksandrov & Beznosikov, 2007 ▸). Atoms *A* rattle in the oversized *X*_12_ icosahedral cage, reducing the lattice component of the thermal conductivity with a minor effect on the electronic properties (Sales *et al.*, 1996 ▸; Nolas *et al.*, 1999 ▸; Keppens *et al.*, 1998 ▸). This feature allows filled skutterudites and other cage compounds to be considered as a promising class of thermoelectric materials.

The proximity of ion bond lengths of transition metals *A* ^6*b*^ and *B* ^8*c*^ with oxygen is another important peculiarity of 1:3 *A*-site-ordered perovskite structure with the 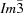 space group. The above peculiarity appears as a result of tilts of octahedra (King & Woodward, 2010 ▸). This suggests the possibility of electronic interactions through *A*′—*X*—*B* chains in addition to the interactions through *B*—*X*—*B* chains that are characteristic of perovskites. As a result, new electronic effects, such as temperature-induced *A*–*B* intersite charge transfer (Long, Hayashi *et al.*, 2009 ▸; Long, Saito *et al.*, 2009 ▸; Zhang *et al.*, 2014 ▸; Long & Shimakawa, 2010 ▸; Yamada *et al.*, 2011 ▸, 2013 ▸, 2016 ▸; Chen *et al.*, 2010 ▸) and a charge disproportionation (Yamada *et al.*, 2008 ▸, 2013 ▸, 2016 ▸) are possible. Yamada *et al.* (2013 ▸), using the example of LnCu_3_Fe_4_O_12_ (Ln = La, Pr, Nd, Sm, Eu, Gd, Tb) perovskites, show that in the case of large Ln ions an intersite charge transfer (3Cu^2+^ + 4Fe^3.75+^ → 3Cu^3+^ + 4Fe^3+^) may arise. However, with smaller Ln ions (Ln = Dy, Ho, Er, Tm Yb, Lu) charge disproportionation (8Fe^3.75+^ → 5Fe^3+^ + 3Fe^5+^) below ∼250−260 K occurs.

From the crystal chemical point of view, the closest to the 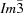 structure is the 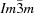 one (Table 2 ▸, Fig. 2 ▸ *a*), which may be represented as an aristotype of the 1:3 *A*-site-ordered perovskites. The structure of the 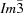 phase differs from the structure of the aristotype in anion positions: the anions in the structure of the 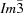 phase occupy the two-parameter 24*g* Wyckoff position with *m* local symmetry, while in the 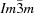 structure the anions occupy the one-parameter 24*h* Wyckoff position with *m*2 local symmetry. Thus, the 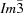 structure can be represented as the 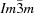 one with the in-phase tilts of anion octahedra characterized by the (φ φ φ) OP that is transformed in accordance with the irrep **k**_11_τ_5_(*M*_2_^+^).

#### 3.2.2. Several OPs   

(i) *Combination of *B*-site ordering and tilts of anion octahedra.* To obtain the list of all possible 1:3 *A*-site-ordered phases resulting from the combination of *B*-site ordering and tilts of anion octahedra, it is necessary to consider the OP which is transformed according to the direct sum of irreps entered into the permutation representation of perovskite structure on the 1*b* Wyckoff position and in the enumeration (2). The permutation representation has the form (Table 1 ▸):
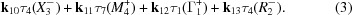



This nine-component OP, transforming according to the direct sum of (2) and (3), generates 174 phases, with only six of them being characterized by 1:3 *A*-site ordering, *B*-site ordering and tilts of anion octahedra (Fig. 1 ▸). Five out of six resulting phases are induced by OPs, in the composition of which irrep **k**_11_τ_7_(*M*_4_^+^) enters. This irrep is associated with 1:3 *B*-site ordering. This case can be seen in Table 2 ▸ (phases 7, 9 and 11). In the formation of phases 8 and 10, apart from the proper OP, improper OPs **k**_13_τ_4_(*R*_2_^−^) and **k**_10_τ_4_(*X*_3_^−^) participate. As a result, more complex 1:1:3:3 *B*-site ordering can be seen in these phases.

**Figure 4:**
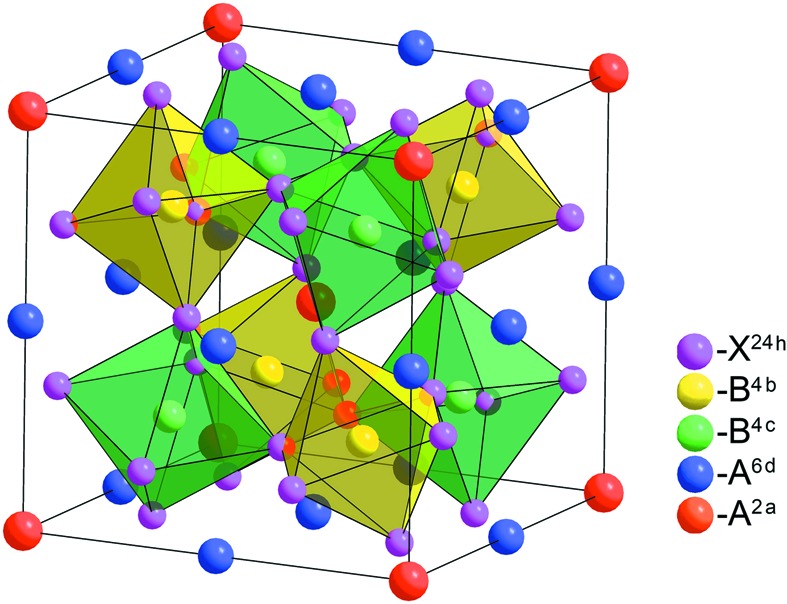
Calculated 1:3 *A*-site-ordered low-symmetry perovskite structures with space group 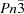.

Let us consider in more detail the 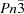 phase formation mechanism (phase 12 of Table 2 ▸), since this phase is observed in a large number of quadruple perovskites, which are sometimes referred to as 1322 perovskites (Senn *et al.*, 2014 ▸). This phase has a structure with eightfold primitive cell volume (Fig. 4 ▸). It is generated by a two-component OP, which is transformed by the direct sum of two irreps **k**_11_τ_5_(*M*_2_^+^) and **k**_13_τ_4_(*R*_2_^−^) [or **k**_11_τ_5_(*M*_2_^+^) and **k**_10_τ_2_(*X*_1_^−^) from the same full set of OPs], linked with the in-phase tilts of anion octahedra and 1:1 *B*-site ordering (rock-salt ordering type). 1:3 *A*-site ordering is generated by an improper OP **k**_11_τ_1_(*M*_1_^+^), which enters into the full set of OPs. This structure can be obtained in two ways from two different intermediate phases: from phase 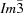 by 1:1 *B*-site ordering generated by the OP **k**_13_τ_4_(*R*_2_^−^) or from phase 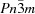 by the tilts of anion octahedra induced by the OP **k**_11_τ_5_(*M*_2_^+^). In the known cases, structural phase transitions in the 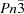 phase pass through the first scenario and are accompanied by a charge disproportionation in the *B* sublattice (Yamada *et al.*, 2008 ▸, 2013 ▸, 2016 ▸; Meng *et al.*, 2017 ▸).

The first synthesized compounds with simultaneous 1:3 ordering in the *A* sublattice and 1:1 rock-salt ordering in the *B* sublattice were CaCu_3_Ga_2_Sn_2_O_12_ and CaCu_3_Ga_2_Ta_2_O_12_ (Byeon *et al.*, 2003 ▸). Later, a similar structure was discovered in CaCu_3_Cr_2_Sb_2_O_12_ (Byeon *et al.*, 2005 ▸), CaCu_3_Fe_2_Sb_2_O_12_ (Chen *et al.*, 2013 ▸) and CaCu_3_Fe_2_Os_2_O_12_ (Deng *et al.*, 2016 ▸). The architecture of this structure allows the exchange interactions between Cu^2+^ and the magnetic *B* cations to be influenced by introducing different cations in the *B*′ sublattice. In addition, the nature of the magnetic properties depends on the degree of ordering in the *B* sublattice, which, as shown by the example CaCu_3_Fe_2_Nb_2_O_12_, varies with different synthesis conditions (Senn *et al.*, 2014 ▸). The compound CaCu_3_Fe_2_Re_2_O_12_ is characterized by semi-metallic behavior with highly spin-polarized conduction electrons, a high Curie temperature and a significant magnetization, which are necessary for spintronic devices (Chen *et al.*, 2014 ▸). Another unusual example of 1322 perovskites is CaCu_3_Fe_4_O_12_, a rare compound containing iron in higher oxidation states (Yamada *et al.*, 2008 ▸). It demonstrates high catalytic activity and stability during oxygen evolution reaction (4OH^−^→O_2_ + 2H_2_O + 4e^−^) (Yagi *et al.*, 2015 ▸).

**Figure 5:**
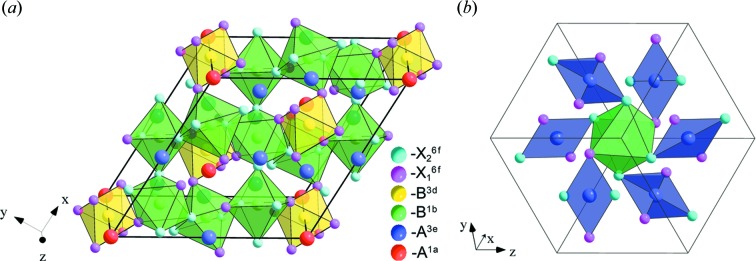
(*a*) Low-symmetry *A*-cation-ordered perovskite structure with space group 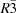. (*b*) 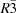 structure in the [111] direction. Only *A* ^3*e*^, *B* ^1^ *^b^* cations and anions are shown. Wyckoff positions are given for trigonal settings.

The calculated structure of phase 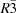 (phase 9 of Table 2 ▸), which is observed in a rich group of ordered perovskites, is presented in Fig. 5 ▸(*a*). CaMn_7_O_12_ (CaMn_3_Mn_4_O_12_) (Bochu *et al.*, 1980 ▸) is one of the compounds with such structure; it demonstrates a giant improper ferroelectricity as a result of the magnetic phase transition to an incommensurate helical magnetic structure below 90 K (Zhang *et al.*, 2011*a* ▸; Johnson *et al.*, 2012 ▸; Perks *et al.*, 2012 ▸). In the phase with multiferroic properties, the direction of the electric polarization axes, the direction of the magnetic helices and MnO_6_ rhombohedra twist coincide (Fig. 5 ▸ *b*). According to Slawinski *et al.* (2009 ▸, 2010 ▸) in solid solutions CaCu*_x_*Mn_7−*x*_O_12_ at *x* = 0 and 0.1, modulation of magnetic moments and also atom displacements below 250 K can be seen.

The 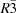 phase formation in CaMn_7_O_12_ occurs during the structural phase transition from the 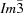 phase with cooling in the range 409–448 K and is accompanied by 1:3 charge ordering of Mn^3+^ and Mn^4+^ ions in the *B* sublattice (Bochu *et al.*, 1980 ▸; Przenioslo *et al.*, 2002 ▸; Troyanchuk & Chobot, 1997 ▸). From the point of view of the Landau theory of phase transitions, such a phase transition can be described by two OPs being transformed by irreps **k**_11_τ_5_(*M*_2_^+^) (in-phase tilts of anion octahedra) and **k**_11_τ_7_(*M*_4_^+^) (cation ordering in the *B* sublattice) (Table 2 ▸) (Howard & Stokes, 2004 ▸). However, a similar structure can be described by distortion of the 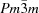 parent structure generated by one OP transformed by irrep **k**_11_τ_9_(*M*_5_^+^) from the same full set of OPs (Fig. 1 ▸, Table 2 ▸). The structural phase transition 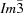
→ 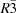 was detected in other manganites: *A*Mn_7_O_12_ (*A* = Pb, Sr, Cd) (Glazkova *et al.*, 2015 ▸; Belik, Glazkova, Terada *et al.*, 2016 ▸). It is interesting to note that in SrMn_7_O_12_ (as well as CaMn_7_O_12_) structural modulations occur below 265 K [**q** = (0, 0, 0.9215) at 113 K] (Belik, Glazkova & Katsuyaal, 2016 ▸). In the case of CdMn_7_O_12_ and PbMn_7_O_12_, lowering of the temperature leads to another structural phase transition to commensurate 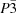 phases at *T*_c_ = 254 K and 294 K, respectively (Belik, Glazkova & Katsuyaal, 2016 ▸; Belik, Glazkova, Terada *et al.*, 2016 ▸; Guo *et al.*, 2017 ▸; Johnson *et al.*, 2017 ▸). These phase transitions to the commensurate phases can be described by adding a third OP which condenses at the point (1/3 1/3 1/3) and is transformed by irrep **k**_9_τ_1_. A rare case of reentrant structural transitions 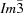 → 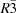 → 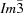, which can be triggered by a magnetic field, was discovered in BiCu*_x_*Mn_7−*x*_O_12_ (0.05 ≤ *x* ≤ 1.1), but its nature remains unknown (Belik, Matsushita & Khalyavin, 2017 ▸).

An unusual form of the mixed metal oxalate KLi_3_Fe(C_2_O_4_)_3_ presented as a perovskite-like structure with 1:3 ordering of both *A* and *B* sites was reported by Yao *et al.* (2017 ▸). In this structure, the Li and Fe atoms are characterized by an octahedral oxygen environment, and the K atoms occupy only one fourth of the *A* sites in the perovskite-like structure positions. Thus, the chemical formula of the compound in a perovskite-like form can be represented as K_1/4_□_3/4_(Li_3/4_Fe_1/4_)(C_1.5_O_3_), where □ is a vacancy. As noted by Yao *et al.* (2017 ▸), this structure is close to the calculated 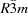 structure generated by the combined atom ordering in *A* and *B* sublattices (phase 8 of Table 2 ▸). However, alternation of oxalate groups in the perovskite structure leads to distortion of anion octahedra and a symmetry change to the 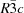 space group (probably phase 11 of Table 2 ▸).

(ii) *Combination of polar displacements and other mechanisms.* In order to obtain a list of all possible 1:3 *A*-site-ordered phases of proper ferroelectrics, it is necessary to choose an OP that transforms in the direct sum of the irrep **k**_12_τ_10_(Γ_4_^−^) connected with the polarization, and the irreps responsible for the realization of other physical mechanisms: orderings in *A*, *B*, *X* sublattices, and tilts of anion octahedra. All low-symmetry phases with 1:3 order in the *A* sublattice, obtained by the above ways, are given in Table 2 ▸ and Fig. 1 ▸.

There are 457 phases induced by the combinations of polarization and tilts and anion octahedra: 391 phases by polarization and *X*-site ordering, 24 phases by polarization and *A*-site ordering. But there are only three different 1:3 *A*-site-ordered polar phases among them. The common factor for all these ferroelectric phases is rhombohedral distortions of the crystal structures and 1:3 *B*-site ordering. The latter is related to the irrep **k**_11_τ_7_(*M*_4_^+^) entering into the full set of OPs for all phases given.

The phases with the *R*3*m* space group (phase 15 of Table 2 ▸) are generated by OPs that are transformed by the direct sum of two irreps, one of which is **k**_12_τ_10_(Γ_4_^−^) and the other **k**_11_τ_1_(*M*_1_^+^) or **k**_11_τ_10_(*M*_5_^−^) or **k**_11_τ_7_(*M*_4_^+^) or **k**_11_τ_9_(*M*_5_^+^). They originate from the combinations of polarization with: proper ordering in the *A* sublattice, ordering in the *X* sublattice, ordering in the *B* sublattice or the tilt-like distortion of the anion octahedra, respectively.

The phases with the space group *R*3 (phases 13 of Table 2 ▸) are generated by the OP which is transformed by the direct sum of two irreps: one of them is **k**_12_τ_10_(Γ_4_^−^) and the other is **k**_11_τ_5_(*M*_2_^+^) or **k**_11_τ_9_(*M*_5_^+^) or **k**_11_τ_10_(*M*_5_^−^) (Aleksandrov & Bartolomé, 2001 ▸; Stokes *et al.*, 2002 ▸; Torgashev *et al.*, 2005 ▸). They originate from the combinations of polarization with *X*-site ordering or one of the two variants of octahedral tilt-like distortions, respectively. The structure of this phase is formed by proper or improper tilts of anion octahedra with rhombohedral displacements of *B* atoms, co-directed with rotation axes (Stokes *et al.*, 2002 ▸). The structure of this phase is observed in the quadruple perovskite Bi_1−*x*/3_(Mn^3+^_3_)(Mn^3+^_4−*x*_Mn^4+^_*x*_)O_12_ in the range 0.10 < *x* < 0.27 (Mezzadri *et al.*, 2011 ▸).

The phase with the *R*3*c* space group (phase 14 of Table 2 ▸) is generated by an OP that is transformed by the direct sum of two irreps **k**_12_τ_10_(Γ_4_^−^) and **k**_10_τ_10_(*X*_5_^−^). The full set of the OPs of this phase includes 19 irreps, which reflect the complexity of this structure.

### 3.3. Some particular cases of phase transitions in 1:3 ordered perovskites   

In this section we will consider the mechanisms of formation of some experimentally observed phases, which were not included in the general classification. The reason for this is either at phase transitions the type of ordering in the *A* sublattice is more complicated than 1:3 (phases with space groups 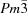, *Pmmm* and *I*2/*m*), or the phase transition is associated with non-polar atom displacements (phases with space group *I*23).

#### 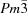 and *Pmmm* phase formation   

3.3.1.

**Figure 6:**
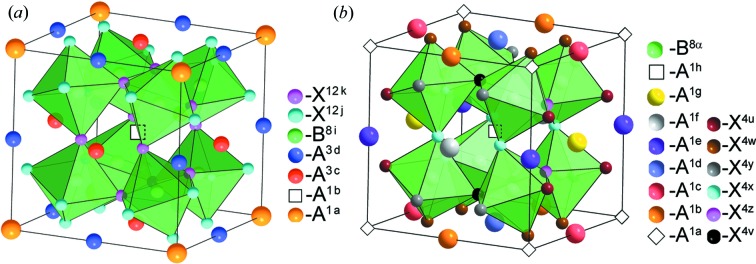
Calculated *A*-site-ordered low-symmetry perovskite structures with space group (*a*) 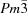 and (*b*) *Pmmm*.

Let us consider the Ce_1/2_Cu_3_Ti_4_O_12_ compound. Depending on the synthesis conditions, this compound exists in two crystalline forms: with random and ordered Ce/vacancy distribution at the *A* site with 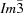 and 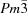 space groups, respectively (Saito *et al.*, 2014 ▸) (Fig. 6 ▸ *a*). Ce/vacancy ordering leads to the appearance of *L*-type ferrimagnetism instead of antiferromagnetism in the disordered structure. Another compound with this structure is CuTa_2_O_6_ (Propach, 1977 ▸), which can be represented as a quadruple perovskite with a cationic deficit: □Cu_2_□Ta_4_O_12_.

The structural mechanisms responsible for the formation of an ordered modification with 1:1:3:3 order in the *A* sublattice differ greatly from those for the disordered phase (Section 3.2.1). The phase structure 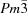 is generated by an OP that is transformed by the direct sum of two irreps: **k**_11_τ_5_(*M*_2_^+^) and **k**_13_τ_1_(*R*_1_^+^). These irreps describe the tilts of anion octahedra (first irrep) and 1:1 *A*-site orderings (second irrep). The full set of the OPs also includes the irreps **k**_11_τ_1_(*M*_1_^+^) (1:3 *A*-site ordering), **k**_10_τ_1_(*X*_1_^+^) (*A*-site ordering), **k**_10_τ_5_(*X*_2_^+^), **k**_12_τ_3_(Γ_2_^+^) and **k**_13_τ_3_(*R*_2_^+^). The combinations of the irreps **k**_12_τ_3_(Γ_2_^+^) and **k**_10_τ_5_(*X*_2_^+^) [or **k**_10_τ_1_(*X*_1_^+^)] from the full set of the OPs can act as the proper OPs. Note that the irrep **k**_10_τ_1_(*X*_1_^+^) is connected with the 1:1:3:3 *A*-site ordering if the direction of the OP vector in OP space is (η η η) (Talanov, Talanov *et al.*, 2014 ▸).

According to Ebbinghaus (2007 ▸), the structure of slow-cooled samples of the nonstoichiometric compound Cu_2+*x*_Ta_4_O_12+σ_ corresponds to the *Pmmm* space group with the splitting of the 1*a* Wyckoff position of the parent phase into eight single Wyckoff positions (only six of them are occupied by copper ions) (Fig. 6 ▸ *b*). As in the case of the 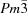 phase, the structural phase transition to this phase can be described by an OP that is transformed by the direct sum of two irreps: **k**_11_τ_5_(*M*_2_^+^) and **k**_13_τ_1_(*R*_1_^+^). But in the case of the *Pmmm* phase, the OP (φ_1_ φ_2_ φ_3_), generated by the irreps **k**_11_τ_5_(*M*_2_^+^), corresponds to another system of tilts of anion octahedra: *a* ^+^ *b* ^+^ *c* ^+^, but not *a* ^+^ *a* ^+^ *a* ^+^ (as in the case of the 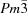 phase) (Howard & Stokes, 1998 ▸). As noted by Ebbinghaus (2007 ▸), the site occupancy of the six different copper sites can vary significantly, depending on the content of copper (*x*) and the cooling speed (slow-cooling or quenching), giving rise to a cooperative vacancy ordering or to a statistical distribution.

#### 3.3.2. *I*23 phase formation   

The structure of LiCuNb_3_O_9_ [the structural formula is (Li_8/3_Cu_8/3_)^6*b*^Nb^8*c*^_8_O^24*f*^_24_] has the space group *I*23 (Sato & Hama, 1993 ▸). In addition, an analogous phase is observed in Fe^2+^- and Ni^2+^-modified CaCu_3_Ti_4_O_12_ (Moriyama *et al.*, 2013 ▸). According to Moriyama *et al.* (2013 ▸), ferroelectric properties are observed in these solid solutions. Concentration phase transition from the centrosymmetric 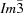 phase into the non-centrosymmetric *I*23 phase is characterized by additional *B*- and *X*-atom displacements (Sato & Hama, 1993 ▸; Moriyama *et al.*, 2013 ▸). There are three irreps **k**_10_τ_1_(*X*_1_^+^), **k**_10_τ_9_(*X*_5_^+^) and **k**_11_τ_6_(*M*_2_^−^) (Table 1 ▸) that enter into mechanical representation on the 1*b* and 3*c* Wyckoff positions of the archetype perovskite structure, but they do not enter into mechanical representation on the 1*a* Wyckoff position (Table 1 ▸). However, only irrep **k**_11_τ_6_(*M*_2_^−^) associated with *B*- and *X*-site non-polar displacement and combined with irrep **k**_11_τ_5_(*M*_2_^+^) induces the phase with the *I*23 space group. Improper OPs enter into the full set of OPs; they are transformed according to the following irreps: **k**_12_τ_2_(Γ_1_^−^), **k**_12_τ_3_(Γ_2_^+^), **k**_12_τ_4_(Γ_2_^−^), **k**_11_τ_1_(*M*_1_^+^) (1:3 *A*-site ordering) and **k**_11_τ_2_(*M*_1_^−^). It is important to note that irrep **k**_12_τ_10_(Γ_4_^−^), according to which the ferroelectric OP (the polarization) is transformed, does not enter into a full set of the OPs. This means that the structural phase transition from 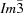 to the *I*23 phase cannot be connected with the ferroelectric structural instabilities.

#### 3.3.3. *I*2/*m* phase formation   

**Figure 7:**
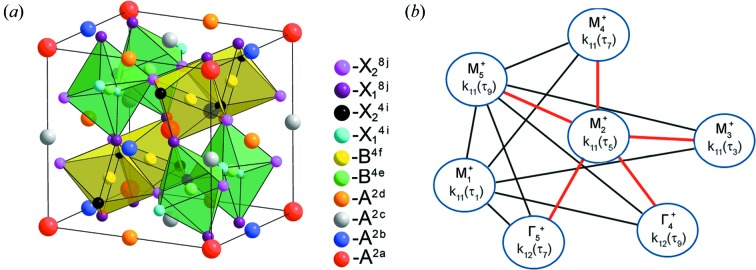
(*a*) Calculated *A*-cation-ordered low-symmetry perovskite structure with space group *I*2/*m*. (*b*) Solid lines indicate pairs of irreps that induce the *I*2/*m* phase formation. Pairs that include **k**_11_τ_5_(*M*_2_^+^) are shown in red.

In LaMn_3_Mn_4_O_12_, a phase transition from 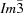 to the *I*2/*m* phase with a 1:1 Mn^3+^/Mn^4+^ charge ordering in the octahedral and 1:1:1 ordering in square-planar positions is observed at *T*_c_ = 653 K (Fig. 7 ▸ *a*). It is accompanied by a change in the system of tilts of anion octahedra from *a* ^+^ *a* ^+^ *a* ^+^ to *a* ^+^ *b* ^+^ *c* ^+^ (Bochu *et al.*, 1974 ▸; Okamoto *et al.*, 2009 ▸). This phase is also observed in *R*Mn_7_O_12_ (*R* = Sm, Eu, Gd and Tb) (Zhang *et al.*, 2018 ▸). In the case of potential multiferroic BiMn_3_Mn_4_O_12_ further cooling leads to *I*2/*m* → *Im* → *P*1 phase transitions at *T* = 460 K and 290 K, respectively (Imamura *et al.*, 2008 ▸; Belik, Matsushita, Kumagai *et al.*, 2017 ▸; Sławiński *et al.*, 2017 ▸; Okamoto *et al.*, 2010 ▸).

The phase transition 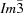 → *I*2/*m* is described by a full set of OPs including the following irreps: **k**_11_τ_1_(*M*_1_^+^), **k**_11_τ_5_(*M*_2_^+^), **k**_11_τ_3_(*M*_3_^+^), **k**_11_τ_7_(*M*_4_^+^), **k**_11_τ_9_(*M*_5_^+^), **k**_12_τ_5_(Γ_3_^+^), **k**_12_τ_9_(Γ_4_^+^), **k**_12_τ_7_(Γ_5_^+^). There are 14 variants of irrep pair combinations, the direct sum of which forms a reducible representation, according to which the proper OP, which induces a low-temperature phase, is transformed (Fig. 7 ▸ *b*). Since it was previously shown that the phase 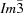 is induced by the OP, which transforms by the irrep **k**_11_τ_5_(*M*_2_^+^), it is necessary to consider the OP as one of the five combinations of irrep **k**_11_τ_5_(*M*_2_^+^) with irreps **k**_12_τ_9_(Γ_4_^+^), **k**_12_τ_7_(Γ_5_^+^), **k**_11_τ_3_(*M*_3_^+^), **k**_11_τ_7_(*M*_4_^+^) and **k**_11_τ_9_(*M*_5_^+^) in order to describe the sequence of 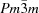 → 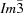 (virtual phase transition) → *I*2/*m* phase transitions. Investigation of the temperature behavior of additional physical parameters, for example, the *C*_44_ elastic modulus [the ferroelastic OP is transformed according to irrep **k**_12_τ_7_(Γ_5_^+^)], the degree of the tilts of anion octahedra [**k**_11_τ_5_(*M*_2_^+^) and **k**_11_τ_9_(*M*_5_^+^)] and others allows one to obtain more detailed information on the phase transition mechanisms.

**Figure 8:**
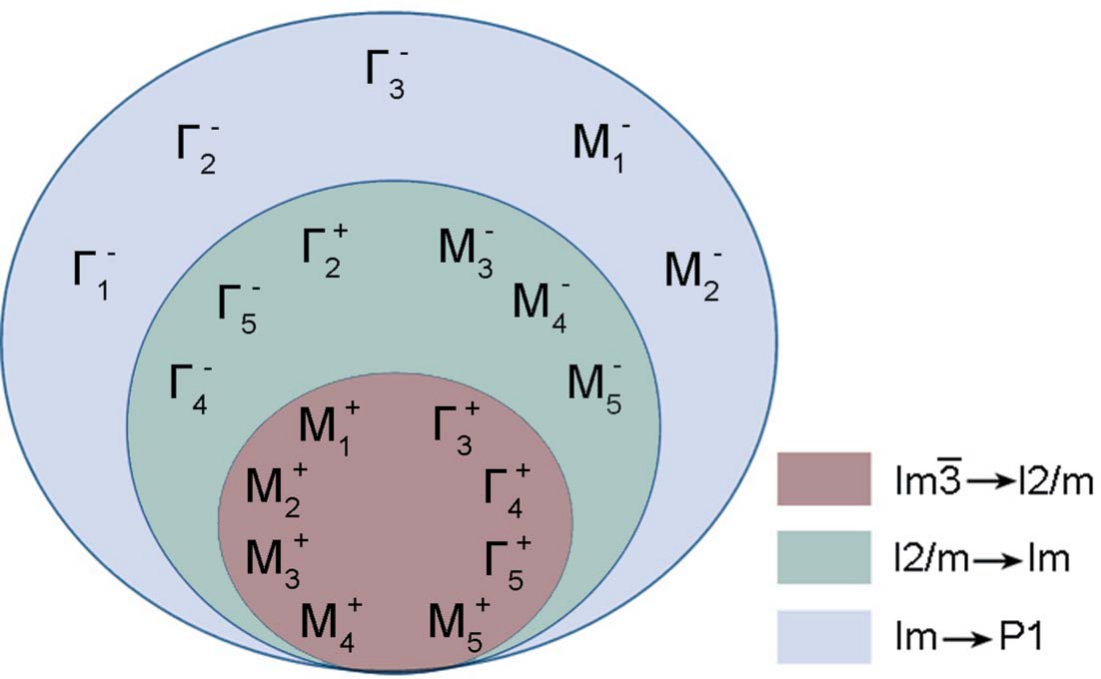
The full set of OPs for the description of the 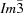 → *I*2/*m* → *Im*→*P*1 phase transition cascade observed in BiMn_3_Mn_4_O_12_.

The phase transition into the polar *Im* phase is described by a full set of the OPs, which also include OPs transforming by the irreps **k**_12_τ_10_(Γ_4_^−^), **k**_12_τ_8_(Γ_5_^−^), **k**_12_τ_3_(Γ_2_^+^), **k**_11_τ_4_(*M*_3_^−^), **k**_11_τ_8_(*M*_4_^−^) and **k**_11_τ_10_(*M*_5_^−^) (Fig. 8 ▸). The entry into the full set of the OPs of the irrep **k**_12_τ_10_(Γ_4_^−^) leads to the possibility of the appearance of ferroelectric properties in this phase. From the crystal chemical point of view, the stabilization of the polar phase is associated with the stereochemical effect induced by the presence of Bi^3+^ ions with the 6*s* ^2^ one pair of electrons (Belik, Matsushita, Kumagai *et al.*, 2017 ▸; Sławiński *et al.*, 2017 ▸; Mezzadri *et al.*, 2009 ▸).

The phase transition to the triclinic *P*1 phase is described by a full set of the OPs, which also includes OPs transforming by the irreps **k**_12_τ_2_(Γ_1_^−^), **k**_12_τ_4_(Γ_2_^−^), **k**_12_τ_6_(Γ_3_^−^), **k**_11_τ_2_(*M*_1_^−^) and **k**_11_τ_6_(*M*_2_^−^) (Fig. 8 ▸). The entry of irrep **k**_11_τ_6_(*M*_2_^−^) into the full set of the OPs leads to additional *B*- and *X*-atom displacements in the perovskite structure.

In another manganite NaMn_7_O_12_ (NaMn_3_Mn_4_O_12_) with Mn^3+^/Mn^4+^ charge ordering, an 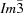 → *I*2/*m* phase transition is observed in the temperature range 168–176 K (Prodi *et al.*, 2004 ▸; Chenavas *et al.*, 1975 ▸). However, further studies have shown that the commensurate modulation with a propagation vector **q** = (1/2, 0, −1/2) can be seen in the low-temperature phase structure of this compound. Thus, the superstructure is described by the *C*2/*m* space group (Prodi *et al.*, 2014 ▸; Streltsov & Khomskii, 2014 ▸).

## 4. Conclusions   

Using group-theoretical methods of the phase transitions theory, possible 1:3 *A*-site-ordered structures are derived from more than 2600 low-symmetry perovskite-like phases obtained by different paths and then analyzed. These phases can be formed from a high-symmetry parent perovskite structure (archetype) as a result of real or virtual structural phase transitions connected with different OPs: atom orderings, atom displacements (polar and non-polar), tilts of octahedra and their combinations. From these phases all 1:3 *A*-site-ordered low-symmetry phases are identified and classified by irreps of the 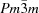 space group (Fig. 1 ▸, Table 2 ▸). Proper and improper OPs, as well as theoretically calculated structures (space groups, multiplications of primitive cells, Wyckoff position splitting) are presented for each of these phases. The structural mechanisms of the formation of some experimentally known 1:3 *A*-site-ordered low-symmetry phases from an archetype perovskite phase have been studied in detail: CaCu_3_Ti_4_O_12_ (the phase structure is characterized by the tilts of anion octahedra), CaCu_3_Ga_2_Sn_2_O_12_ (the phase structure can be represented by a combination of the tilts of anion octahedra and 1:1 *B*-site ordering), CaMn_3_Mn_4_O_12_ (the structure of the phase can be considered as a combination of the tilts of anion octahedra and 1:3 *B*-site ordering), Ce_1/2_Cu_3_Ti_4_O_12_ (the phase structure is characterized by a combination of tilts of anion octahedra and additional 1:1 *A*-site ordering) and others.

**Figure 9:**
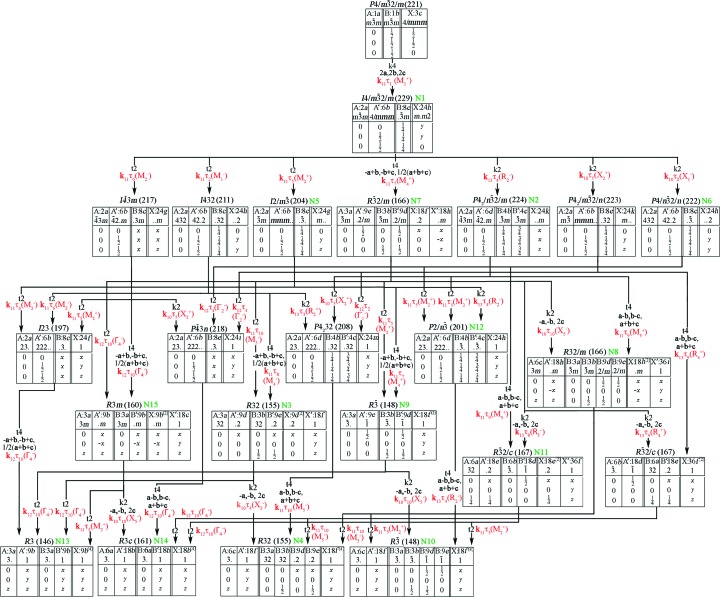
Modified Bärnighausen tree of all possible 1:3 *A*-site-ordered low-symmetry phases which was constructed using the ITC approach with the same limitations as in the R-approach (2*a*, 2*b*, 2*c* maximal primitive cell, only commensurate phases and 1:3 splitting type of 1*a* Wyckoff position of archetype structure). The irreps are designated in red. The numbers of phases from Table 2 ▸ are designated in green. All translations of the primitive cells of the low-symmetry phases in this figure relate to the primitive cell of the aristotype phase, which may vary for different phases of the hettotypes. In Table 2 ▸, all translations are given relative to the primitive cell of a parent perovskite structure (archetype) that is common to all low-symmetry phases.

Fig. 9 ▸ shows a modified Bärnighausen tree of all possible 1:3 *A*-site-ordered low-symmetry phases, based on the ITC approach (ITC, 2010 ▸; Müller, 2013 ▸) with the irreps indicated, describing relative phase transitions. Note the fundamental peculiarity of convergence of two approaches in the modified Bärnighausen tree. All group–subgroup relations when using the ITC approach are represented by different pairs of aristo­type and hettotype phases.

In the case of the R-approach, irreps describe all the low-symmetry phases that occur during a real or virtual phase transition from only one unchanged archetype phase, regardless of the intermediate phases. This is an important distinction. It significantly complicates the representation of the paths of formation of low-symmetry phases and the visualization of the results obtained. Therefore, the number of OPs describing the formation of some low-symmetry phases (Fig. 9 ▸) is not the minimum number of OPs. For example, from the modified Bärnighausen tree several OPs [**k**_11_τ_1_(*M*_1_^+^), **k**_11_τ_7_(*M*_4_^+^), **k**_13_τ_8_(*R*_5_^−^), **k**_10_τ_2_(*X*_1_^−^), **k**_11_τ_9_(*M*_5_^+^)] can describe the formation of the phase with space group 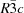 (phase 11, Table 2 ▸) depending on the path. In the case of the R-approach, the same phase can be obtained from the structure of the archetype by only one of its proper OPs, which are transformed by irrep **k**_10_τ_10_(*X*_5_^−^). However, the combination of the two approaches allows us to track the formation of a full set of OPs, which is unchanged for each of the phases regardless of the path, and fully determines its structure.

Note that all the phases obtained using the R-approach are presented in Fig. 1 ▸ and Table 2 ▸, as well as Fig. 9 ▸. But in Fig. 9 ▸, there are several phases that were not obtained by using the R-approach. These are phases with space groups 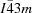, *I*432, 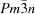, 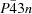 and *P*4_2_32. Let us explain this inconsistency in the results obtained by the R- and ITC approaches. The program *COPL* (Stokes & Hatch, 2002 ▸; Hatch & Stokes, 2002 ▸) helped us to ascertain that the first three phases are induced by OPs, which are transformed by irreps **k**_11_τ_6_(*M*_2_^−^), **k**_11_τ_2_(*M*_1_^−^) and **k**_10_τ_5_(*X*_2_^+^), respectively. Irrep **k**_11_τ_6_(*M*_2_^−^) enters into mechanical representation on Wyckoff positions 1*b* and 3*c* of the perovskite structure (Table 1 ▸). This irrep induces non-polar displacements of *B* and *X* atoms and, therefore, it has not been considered. Irreps **k**_11_τ_2_(*M*_1_^−^) and **k**_10_τ_5_(*X*_2_^+^) enter neither into permutational nor mechanical representations of the perovskite structure. According to Boström *et al.* (2018 ▸) OPs transforming by irreps **k**_11_τ_6_(*M*_2_^−^) and **k**_10_τ_5_(*X*_2_^+^) are associated with columnar shifts and quadrupolar *A*-site order in molecular perovskites. The phases with space groups 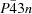 and *P*4_2_32 can be induced by the combination of the OPs **k**_12_τ_4_(Γ_2_^−^) and **k**_10_τ_5_(*X*_2_^+^) in the first case, and **k**_11_τ_2_(*M*_1_^−^) and **k**_13_τ_3_(*R*_2_^+^) or **k**_12_τ_2_(Γ_1_^−^) and **k**_10_τ_5_(*X*_2_^+^) in the second case. The physical nature of these parameters is unknown. The 1:3 *A*-site ordering in all five cases is explained by the occurrence of the improper OP transformed by irrep **k**_11_τ_1_(*M*_1_^+^) in the full set of OPs. Thus, the five phases induced by the indicated irreps are beyond the limitations considered (Fig. 1 ▸). Besides, note the existence of two similar phases with the same space group 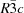, but with the different splittings of Wyckoff positions. Atom Wyckoff positions from the *A* sublattice of the first phase correspond to atom Wyckoff positions from the *B* sublattice of the second phase and vice versa. This is due to the internal automorphism of the positions 1*a* and 1*b* in the perovskite structure. Thus, the structure of one of the phases is induced by the OP transformed by irrep **k**_13_τ_9_(*R*_4_^+^), which is equivalent to the irrep **k**_13_τ_8_(*R*_5_^−^) in the case when the origin is at the 1*b* position. But when the origin is at the 1*a* position, as in this work, the physical meaning of this OP is unclear, and that is why irrep **k**_13_τ_9_(*R*_4_^+^) has not been considered.

Fundamentally important is the following result. Most of the hettotype phases presented here can be obtained in various ways from different aristotype phases (Fig. 9 ▸). For example, the phase with space group 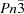 (hettotype) can be obtained from the aristotype phases 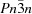, 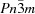 or 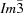 by *t*2, by *t*2 or by *k*2, respectively, which, in turn, can be obtained from the phase with the space group 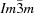 by *k*2, by *k*2 or by *t*2, respectively (Figs. 9 ▸, 10 ▸). A similar situation arises in the variability found in selecting proper OPs from the full set of OPs in the R-approach (Fig. 1 ▸, Table 2 ▸). The same phase with the space group 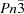 can be induced from the phase with the space group 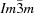 by three paths: in-phase tilts of octahedra [irrep **k**_11_τ_5_(*M*_2_^+^)] and *B*-site ordering [irrep **k**_13_τ_4_(*R*_2_^−^)] via the intermediate 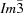 phase, or the reverse sequence of the same OPs via the intermediate 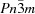 phase or tilt-like distortions [irrep **k**_10_τ_2_(*X*_1_^−^)] and in-phase tilts of octahedra [irrep **k**_11_τ_5_(*M*_2_^+^)] via the intermediate 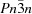 phase. As stated above (Section 3.2.2), the known examples are realizations of the first scenario only (phase 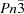 is formed by *B*-site ordering). It should be noted that, regardless of the path of phase genesis, the full set of OPs remains unchanged.

The generalization of the result obtained is the principle of variation in selecting the paths for group-theoretical design of new phases. This principle can be formulated as follows: the same low-symmetry phase (hettotype) can be obtained from the high-symmetry phase (archetype) in several ways depending on the selection of the proper OPs from the full set of OPs, with the space-group type of the low-symmetry phase and the splitting of Wyckoff positions remaining the same, regardless of the genesis path of the low-symmetry phase.

An even more complicated case is observed during the description of the 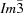 → *I*2/*m* → *Im* → *P*1 phase transition cascade which occurs in BiMn_3_Mn_4_O_12_. At each step of the cascade, the number of irreps entering into a full set of the OPs increases. As a result, the number of options for choosing proper OPs in order to describe the entire sequence of structural phase transitions also increases. Thus, this is a first presentation of the variability in the choice of the proper OPs within the unchanged full set of OPs. This result emphasizes the need to consider the full set of OPs rather than proper OPs only. The observed variability in the choice of the proper OPs, which generate 1:3 *A*-site-ordered low-symmetry phases, highlights the fundamental significance of the full set of OPs when describing the structural phase transitions. The variability in the choice of the proper OPs within the unchanged full set of OPs is a new phenomenon revealed in our study. This extraordinary and important phenomenon requires further research.

Thus, using the example of 1:3 *A*-site-ordered perovskites, this study showed that the combined use of ITC and R-approaches allows determination of OPs that describe the structural phase transitions (including virtual transitions) and the structures of low-symmetry phases as well as investigation of the structural genesis and group–subgroup relations between different crystalline phases/structures.

## Figures and Tables

**Figure 10:**
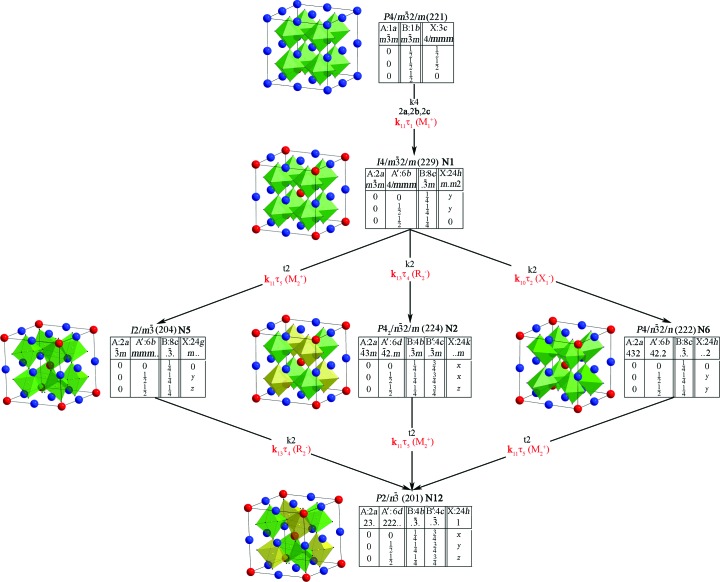
Possible group-theoretical paths for obtaining a phase with the space group 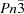 by the ITC and R-approaches.

**Table 1:** Physical realization of the OP connected with atom non-polar and polar displacements, atom orderings and tilts of octahedra in perovskite structure (the unit irrep is not shown)

	Wavevectors			
Physical realization	**k**_10_(0 0 ½)(*X*)	**k**_11_(0 ½ ½)(*M*)	**k**_12_(0 0 0)(Γ)	**k**_13_(½ ½ ½)(*R*)
**Non-polar displacement**				
*A*-site (*D*_*A*_)	τ_4_(*X*_3_^−^) + τ_10_(*X*_5_^−^)	τ_4_(*M*_3_^−^) + τ_10_(*M*_5_^−^)		τ_10_(*R*_4_^−^)
*B*-site (*D*_*B*_)	τ_1_(*X*_1_^+^) + τ_9_(*X*_5_^+^)	τ_6_(*M*_2_^−^) + τ_10_(*M*_5_^−^)		τ_7_(*R*_5_^+^)
*X*-site (*D*_*X*_)	τ_1_(*X*_1_^+^) + τ_4_(*X*_3_^−^) + τ_6_(*X*_2_^−^) + 2τ_9_(*X*_5_^+^) + τ_10_(*X*_5_^−^)	τ_1_(*M*_1_^+^) + τ_3_(*M*_3_^+^)+ τ_5_(*M*_2_^+^) + τ_6_(*M*_2_^−^) + τ_7_(*M*_4_^+^) + τ_9_(*M*_5_^+^)+ τ_10_(*M*_5_^−^)	τ_8_(Γ_5_^−^)	τ_1_(*R*_2_^−^) + τ_6_(*R*_3_^−^) + τ_8_(*R*_5_^−^) + τ_10_(*R*_4_^−^)
				
**Polar displacement**				
*A*-, *B*-, *X*-sites (*P*)			τ_10_(Γ_4_^−^)	
				
**Ordering**				
*A*-site (*O*_*A*_)	τ_1_(*X*_1_^+^)	τ_1_(*M*_1_^+^)	τ_1_(Γ_1_^+^)	τ_1_(*R*_1_^+^)
*B*-site (*O*_*B*_)	τ_4_(*X*_3_^−^)	τ_7_(*M*_4_^+^)	τ_1_(Γ_1_^+^)	τ_4_(*R*2^−^)
*X*-site (*O*_*X*_)	τ_1_(*X*_1_^+^) + τ_4_(*X*_3_^−^) + τ_8_(*X*_4_^−^)	τ_7_(*M*_4_^+^) + τ_10_(*M*_5_^−^)	τ_1_(Γ_1_^+^) + τ_5_(Γ_3_^+^)	τ_7_(*R*_5_^+^)
				
**Tilts or tilt-like distortions**				
*B*-site (*V*_*B*_)	τ_2_(*X*_1_^−^) + τ_10_(*X*_5_^−^)	τ_5_(*M*_2_^+^) + τ_9_(*M*_5_^−^)	τ_9_(Γ_4_^+^)	τ_8_(*R*_5_^−^)

**Table 2:** Possible low-symmetry 1:3 *A*-site-ordered perovskite phases characterized by different types of OPs The following designations of OPs are used: **k**_10_(*X*) = η, **k**_11_(*M*) = φ, **k**_12_(Γ) = σ and **k**_13_(*R*) = Ψ. In the second and third columns, the directions of OPs in OP space are given. Then notations of the irreps according to Kovalev (1993 ▸) are provided. The designations of the irreps by Miller & Love (1967 ▸) used in the *ISOTROPY* program are given in parentheses. The symbols *V*, *O*, *P* and *D* denote the physical nature of OPs associated with tilts of anion octahedra (or tilt-like distortions), atom orderings, and polar and non-polar atom displacements, respectively. The type of perovskite sublattice in which the OP is implemented is indicated by a subscript. The unit irrep **k**_12_(τ_1_)(Γ_1_^+^) is not shown. *V*/*V*_0_ is the multiplication of the primitive cell volume as a result of virtual structural phase transition from the archetype structure. The superscript index in the structural formula means the type of Wyckoff position according to the *International Tables for Crystallography*.

No.	Proper OP	Improper OP	Space group	Subgroup basis/origin	*V*/*V*_0_	Structural formula
**Proper 1:3 *A*-site ordering**						
1	(φ φ φ); **k**_11_τ_1_(*M*_1_^+^); *O*_*A*_ *D*_*X*_		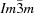 (No. 229)	2**a**_1_, 2**a**_2_, 2**a**_3_ (0,0,0)	4	*A* ^2*a*^_1/4_ *A* ^6*b*^_3/4_ *B* ^8*c*^ *X* ^24*h*^_3_
						
***A*+*B*-site ordering**						
2	(φ φ φ); **k**_11_τ_1_(*M*_1_^+^); *O*_*A*_ *D*_*X*_ Ψ; **k**_13_τ_4_(*R*_2_^−^); *O*_*B*_ *D*_*X*_	(η η η);**k**_13_τ_6_(*X*_2_^−^);*D*_*X*_	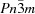 (No. 224)	−2**a**_2_, −2**a**_1_, −2**a**_3_ (3/2,3/2,3/2)	8	*A* ^2*a*^_1/4_ *A* ^6*d*^_3/4_ *B* ^4*b*^_1/2_ *B* ^4*c*^_1/2_ *X* ^24*k*^_3_
						
***X*-site ordering**						
3	(φ −φ φ −φ φ −φ); **k**_11_τ_10_(*M*_5_^−^); *O*_*X*_ *D*_*A*_ *D*_*B*_ *D*_*X*_	(φ φ φ); **k**_11_τ_1_(*M*_1_^+^); *O*_*A*_ *D_X_*; (φ φ φ); **k**_11_τ_2_(*M*_1_^−^); (φ φ φ); **k**_11_τ_7_(*M*_4_^+^); *O_B_* *O_X_* *D_X_*; (φ φ φ); **k**_11_τ_8_(*M*_4_^−^); (φ −φ φ −φ φ −φ); **k**_11_τ_9_(*M*_5_^+^); *V_B_* *D_X_*; σ; **k**_12_τ_2_(Γ_1_^−^); (σ σ σ); **k**_12_τ_7_(Γ_5_^+^); (σ σ σ); **k**_12_τ_8_(Γ_5_^−^); *D_X_*	*R*32 (No. 155)	2**a**_1_−2**a**_3_, −2**a**_2_+2**a**_3_, −**a**_1_−**a**_2_−**a**_3_ (0,0,0)	4	*A* ^3*a*^_1/4_ *A* ^9*d*^_3/4_ *B* ^3*b*^_1/4_ *B* ^9*e*^_3/4_ *X* ^18*f*^_6/4_ *X* ^9*d*(2)^_6/4_
4	(φ −φ φ −φ φ −φ); **k**_11_τ_10_(*M*_5_^−^); *O_X_* *D_A_* *D_B_* *D_X_* (η η η); **k**_10_τ_4_(*X*_3_^−^); *O_B_* *O_X_* *D_A_* *D_X_*	(η η η); **k**_10_τ_5_(*X*_2_^+^); (η η η); **k**_10_τ_3_(*X*_3_^+^); *O_X_*; (η η η η η η); **k**_10_τ_9_(*X*_5_^+^); *D_B_* *D_X_*; (η η η); **k**_10_τ_6_(*X*_2_^−^); *D_X_*; (η η η η η η); **k**_10_τ_10_(*X*_5_^−^); *V_B_* *D_A_* *D_X_*; (φ φ φ); **k**_11_τ_1_(*M*_1_^+^); *O_A_* *D_X_*; (φ φ φ); **k**_11_τ_2_(*M*_1_^−^); (φ φ φ); **k**_11_τ_7_(*M*_4_^+^); *O_B_* *O_X_* *D_X_*; (φ φ φ); **k**_11_τ_8_(*M*_4_^−^); (φ −φ φ −φ φ −φ); **k**_11_τ_9_(*M*_5_^+^); *V_B_* *D_X_*; σ; **k**_12_τ_2_(Γ_1_^−^); (σ σ σ); **k**_12_τ_8_(Γ_5_^−^); *D_X_*; (σ σ σ); **k**_12_τ_7_(Γ_5_^+^); Ψ; **k**_13_τ_3_(*R*_2_^+^); (Ψ Ψ Ψ); **k**_13_τ_9_(*R*_4_^+^); Ψ; **k**_13_τ_4_(*R*_2_^−^); *O_B_* *D_X_*; (Ψ Ψ Ψ); **k**_13_τ_10_(*R*_4_^−^); *D_A_* *D_X_*	*R*32 (No. 155)	−2**a**_1_+2**a**_3_, 2**a**_2_−2**a**_3_, −2**a**_1_−2**a**_2_−2**a**_3_ (−1/2,−1/2,−1/2)	8	*A* ^6*c*^_1/4_ *A* ^18*f*^_3/4_ *B^3^* ^a^_1/8_ *B* ^3*b*^_1/8_ *B* ^9*d*^_3/8_ *B* ^9*e*^_3/8_ *X* ^18*f*(4)^_3_
						
**Tilts of anion octahedra or tilt-like distortions**						
5	(φ φ φ); **k**_11_τ_5_(*M*_2_^+^); *V_B_* *D_X_*	(φ φ φ); **k**_11_τ_1_(*M*_1_^+^); *O_A_* *D_X_*; σ; **k**_12_τ_3_(Γ_2_^+^)	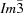 (No. 204)	2**a**_1_, 2**a**_2_, 2**a**_3_ (0,0,0)	4	*A* ^2*a*^_1/4_ *A* ^6*b*^_3/4_ *B* ^8*c*^ *X* ^24*g*^_3_
6	(η η η); **k**_10_τ_2_(*X*_1_^−^); *V_B_*	(φ φ φ); **k**_11_τ_1_(*M*_1_^+^); *O_A_* *D_X_*;* Ψ*; **k**_13_τ_2_(*R*_1_^−^)	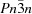 (No. 222)	2**a**_1_, 2**a**_2_, 2**a**_3_ (1/2,3/2,3/2)	8	*A* ^2*a*^_1/4_ *A* ^6*b*^_3/4_ *B* ^8*c*^ *X* ^24*h*^_3_
7	(φ −φ φ −φ φ −φ); **k**_11_τ_9_(*M*_5_^+^); *V_B_* *D_X_* or (φ φ φ); **k**_11_τ_1_(*M*_1_^+^); *O_A_* *D_X_* (φ φ φ); **k**_11_τ_7_(*M*_4_^+^); *O_B_* *O_X_* *D_X_*	(φ φ φ); **k**_11_τ_1_(*M*_1_^+^); *O_A_* *D_X_*; (φ φ φ); **k**_11_τ_7_(*M*_4_^+^); *O_B_* *O_X_* *D_X_*; (φ −φ φ −φ φ −φ); **k**_11_τ_9_(*M*_5_^+^); *V_B_* *D_X_*; (σ σ σ); **k**_12_τ_7_(Γ_5_^+^)	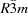 (No. 166)	2**a**_1_−2**a**_3_, −2**a**_2_+2**a**_3_, −**a**_1_−**a**_2_−**a**_3_ (0,0,0)	4	*A* ^3*a*^_1/4_ *A* ^9*e*^_3/4_ *B* ^3*b*^_1/4_ *B* ^9*d*^_3/4_ *X* ^18*h*^_3/2_ *X* ^18*f*^_3/2_
8	(η η η η η η); **k**_10_τ_10_(*X*_5_^−^); *V_B_* *D_A_* *D_X_* or (φ φ φ); **k**_11_τ_1_(*M*_1_^+^); *O_A_* *D_X_* (η η η); **k**_11_τ_4_(*X*_3_^−^); *O_B_* *D_A_* *D_X_*	(η η η); **k**_10_τ_6_(*X*_2_^−^); *D_X_*; (η η η); **k**_10_τ_4_(*X*_3_^−^); *O_B_* *O_X_* *D_A_* *D_X_*; (η η η η η η); **k**_10_τ_10_(*X*_5_^−^); *V_B_* *D_A_* *D_X_*; (φ φ φ); **k**_11_τ_1_(*M*_1_^+^); *O_A_* *D_X_*; (φ φ φ); **k**_11_τ_7_(*M*_4_^+^); *O_B_* *O_X_* *D_X_*; (φ −φ φ −φ φ −φ); **k**_11_τ_9_(*M*_5_^+^); *V_B_* *D_X_*; (σ σ σ); **k**_12_τ_7_(Γ_5_^+^); Ψ; **k**_13_τ_4_(*R*_2_^−^); *O_B_* *D_X_*; (Ψ Ψ Ψ); **k**_13_τ_10_(*R*_4_^−^); *D_A_* *D_X_*	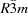 (No. 166)	−2**a**_1_+2**a**_3_, 2**a**_2_−2**a**_3_, −2**a**_1_−2**a**_2_−2**a**_3_ (−1/2,−1/2,−1/2)	8	*A* ^6*c*^_1/4_ *A* ^18*h*^_3/4_ *B* ^3*a*^_1/8_ *B* ^3*b*^_1/8_ *B* ^9*d*^_3/8_ *B* ^9*e*^_3/8_ *X* ^18*h*(2)^_3/2_ *X* ^36*i*^_3/2_
9	(φ_1_ φ_2_ φ_1_ φ_2_ φ_1_ φ_2_); **k**_11_τ_9_(*M*_5_^+^);*V_B_* *D_X_* or (φ φ φ); **k**_11_τ_5_(*M*_2_^+^); *V_B_* *D_X_* (φ φ φ); **k**_11_τ_7_(*M*_4_^+^); *O_B_* *O_X_* *D_X_*	(φ φ φ);**k**_11_τ_1_(*M*_1_^+^);*O_A_* *D_X_*; (φ φ φ);**k**_11_τ_3_(*M*_3_^+^);*D_X_*; (φ φ φ);**k**_11_τ_5_(*M*_2_^+^);*V_B_* *D_X_*; (φ φ φ);**k**_11_τ_7_(*M*_4_^+^);*O_B_* *O_X_* *D_X_*; (φ_1_ φ_2_ φ_1_ φ_2_ φ_1_ φ_2_); **k**_11_τ_9_(*M*_5_^+^);*V_B_* *D_X_*; σ;**k**_12_τ_3_(Γ_2_^+^); (σ σ σ);**k**_12_τ_7_(Γ_5_^+^); (σ σ σ);**k**_12_τ_9_(Γ_4_^+^);*V_B_*	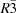 (No. 148)	−2**a**_1_+2**a**_2_, −2**a**_2_+2**a**_3_, **a**_1_+**a**_2_+**a**_3_ (0,0,0)	4	*A* ^3*a*^_1/4_ *A* ^9*e*^_3/4_ *B* ^3*b*^_1/4_ *B* ^9*d*^_3/4_ *X* ^18*f*(2)^_3_
10	(η_1_ η_2_ η_1_ η_2_ η_1_ η_2_); **k**_10_τ_10_(*X*_5_^−^); *V_B_* *D_A_* *D_X_* or (η_1_ η_2_ η_1_ η_2_ η_1_ η_2_); **k**_10_τ_10_(*X*_5_^−^); *V*_B_ *D*_A_ *D*_X_ (φ φ φ); **k**_11_τ_7_(*M*_4_^+^); *O*_B_ *O*_X_ *D*_X_	(η η η); **k**_10_τ_2_(*X*_1_^−^); *V_B_*; (η η η); **k**_10_τ_4_(*X*_3_^−^); *O_B_* *O_X_* *D_A_* *D_X_*; (η η η); **k**_10_τ_6_(*X*_2_^−^); *D_X_*; (η η η); **k**_10_τ_8_(*X*_4_^−^); *O_X_*; (φ φ φ); **k**_11_τ_1_(*M*_1_^+^); *O_A_* *D_X_*; (φ φ φ); **k**_11_τ_3_(*M*_3_^+^); *D_X_*; (φ φ φ); **k**_11_τ_5_(*M*_2_^+^); *V_B_* *D_X_*; (φ φ φ); **k**_11_τ_7_(*M*_4_^+^); *O_B_* *O_X_* *D_X_*; (φ_1_ φ_2_ φ_1_ φ_2_ φ_1_ φ_2_); **k**_11_τ_9_(*M*_5_^+^); *V_B_* *D_X_*; σ; **k**_12_τ_3_(Γ_2_^+^); (σ σ σ); **k**_12_τ_7_(Γ_5_^+^); (σ σ σ); **k**_12_τ_9_(Γ_4_^+^); *V_B_*; Ψ; **k**_13_τ_2_(*R*_1_^−^); Ψ;**k**_13_τ_4_(*R*_2_^−^); *O_B_* *D_X_*; (Ψ Ψ Ψ); **k**_13_τ_8_(*R*_5_^−^); *V_B_* *D_X_*; (Ψ Ψ Ψ); **k**_13_τ_10_(*R*_4_^−^); *D_A_* *D_X_*	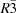 (No. 148)	2**a**_2_−2**a**_3_, 2**a**_1_−2**a**_2_, −2**a**_1_−2**a**_2_−2**a**_3_ (−1/2,−1/2,−1/2)	8	*A* ^6*c*^_1/4_ *A* ^18*f*^_3/4_ *B* ^3*a*^_1/8_ *B* ^9*e*^_3/8_ *B* ^9*d*^_3/8_ *B* ^3*b*^_1/8_ *X* ^18*f*(4)^_3_
11	(η −η η −η η −η); **k**_10_τ_10_(*X*_5_^−^); *V_B_* *D_A_* *D_X_* or (Ψ Ψ Ψ); **k**_13_τ_8_(*R*_5_^−^); *V_B_* *D_X_* (φ φ φ); **k**_11_τ_7_(*M*_4_^+^); *O_B_* *O_X_* *D_X_*	(η η η); **k**_10_τ_2_(*X*_1_^−^); *V_B_*; (η η η); **k**_10_τ_8_(*X*_4_^−^); *O_X_*; (η −η η −η η −η); **k**_10_τ_10_(*X*_5_^−^); *V_B_* *D_A_* *D_X_*; (φ φ φ); **k**_11_τ_1_(*M*_1_^+^); *O_A_* *D_X_*; (φ φ φ); **k**_11_τ_7_(*M*_4_^+^); *O_B_* *O_X_* *D_X_*; (φ −φ φ −φ φ −φ); **k**_11_τ_9_(*M*_5_^+^); *V_B_* *D_X_*; (σ σ σ); **k**_12_τ_7_(Γ_5_^+^); Ψ; **k**_13_τ_2_(*R*_1_^−^); (Ψ Ψ Ψ); **k**_13_τ_8_(*R*_5_^−^); *V_B_* *D_X_*	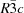 (No. 167)	−2**a**_1_+2**a**_3_, 2**a**_2_−2**a**_3_, −2**a**_1_−2**a**_2_−2**a**_3_ (−1/2,−1/2,−1/2)	8	*A* ^6*a*^_1/4_ *A* ^18***e***^_3/4_ *B* ^6*b*^_1/4_ *B* ^18*d*^_3/4_ *X* ^36*f*^_6/4_ *X* ^18*e*(2)^_6/4_
						
***B*-site ordering and tilts of anion octahedra**						
12	(φ φ φ); **k**_11_τ_5_(*M*_2_^+^); *V_B_* *D_X_* Ψ; **k**_13_τ_4_(*R*_2_^−^); *O_B_* *D_X_* or (φ φ φ); **k**_11_τ_5_(*M*_2_^+^); *V_B_* *D_X_* (η η η); **k**_10_τ_2_(*X*_1_^−^); *V_B_*	(η η η); **k**_10_τ_2_(*X*_1_^−^); *V_B_*; (η η η); **k**_10_τ_6_(*X*_2_^−^); (φ φ φ); **k**_11_τ_1_(*M*_1_^+^); *O_A_* *D_X_*; σ; **k**_12_τ_3_(Γ_2_^+^); Ψ; **k**_13_τ_2_(*R*_1_^−^); Ψ; **k**_13_τ_4_(*R*_2_^−^); *O_B_* *D_X_*	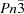 (No. 201)	−2**a**_3_, −2**a**_2_, −2**a**_1_ (1/2,1/2,1/2)	8	*A* ^2*a*^_1/4_ *A* ^6*d*^_3/4_ *B* ^4*c*^_1/2_ *B* ^4*b*^_1/2_ *X* ^24*h*^_3_
						
**Polar displacements**						
13	(φ_1_ φ_2_ φ_1_ φ_2_ φ_1_ φ_2_);**k**_11_τ_10_(*M*_5_^−^);*O_X_* *D_A_* *D_B_* *D_X_* or (σ σ σ); **k**_11_τ_10_(Γ_4_^−^); P (φ φ φ); **k**_11_τ_5_(*M*_2_^+^); *V*_B_ *D*_X_	(φ φ φ); **k**_11_τ_1_(*M*_1_^+^); *O_A_* *D_X_*; (φ φ φ); **k**_11_τ_2_(*M*_1_^−^); (φ φ φ); **k**_11_τ_3_(*M*_3_^+^); *D_X_*; (φ φ φ); **k**_11_τ^4^(*M*_3_^−^); *D_A_*; (φ φ φ); **k**_11_τ_5_(*M*_2_^+^); *V_B_* *D_X_*; (φ φ φ); **k**_11_τ_6_(*M*_2_^−^); *D_B_* *D_X_*; (φ φ φ); **k**_11_τ_7_(*M*_4_^+^); *O_B_* *O_X_* *D_X_*; (φ φ φ); **k**_11_τ_8_(*M*_4_^−^); (φ_1_ φ_2_ φ_1_ φ_2_ φ_1_ φ_2_); **k**_11_τ_9_(*M*_5_^+^); *V_B_* *D_X_*; (φ_1_ φ_2_ φ_1_ φ_2_ φ_1_ φ_2_); **k**_11_τ_10_(*M*_5_^−^); *O_X_* *D_A_* *D_B_* *D_X_* σ; **k**_12_τ_2_(Γ_1_^−^); σ; **k**_12_τ_3_(Γ_2_^+^); σ; **k**_12_τ_4_(Γ_2_^−^); (σ σ σ); **k**_12_τ_7_(Γ_5_^+^); (σ σ σ); **k**_12_τ_8_(Γ_5_^−^); *D_X_*; (σ σ σ); **k**_12_τ_9_(Γ_4_^+^); *V_B_*	*R*3 (No. 146)	−2**a**_1_+2**a**_2_, 2**a**_1_−2**a**_3_, −**a**_1_ −**a**_2_−**a**_3_ (0,0,0)	4	*A* ^3*a*^_1/4_ *A* ^9*b*^_3/4_ *B* ^3*a*^_1/4_ *B* ^9*b*^_3/4_ *X* ^9*b*(4)^_3_
14	(σ σ σ); **k**_11_τ_10_(Γ_4_^−^); *P* (η −η η η η η); **k**_10_τ_10_(*X*_5_^−^); *V_B_* *D_A_* *D_X_*	(η η η); **k**_10_τ_2_(*X*_1_^−^); *V_B_*; (η η η); **k**_10_τ_3_(*X*_3_^+^); *O_X_*; (η η η); **k**_10_τ_5_(*X*_2_^+^); (η η η); **k**_10_τ_8_(*X*_4_^−^); *O_X_*; (η η η η η η); **k**_10_τ_9_(*X*_5_^+^); *D_B_* *D_X_*; (φ φ φ); **k**_11_τ_1_(*M*_1_^+^); *O_A_* *D_X_*; (φ φ φ); **k**_11_τ_4_(*M*_3_^−^); *D_A_*; (φ φ φ); **k**_11_τ_6_(*M*_2_^−^); *D_B_* *D_X_*; (φ φ φ); **k**_11_τ_7_(*M*_4_^+^); *O_B_* *O_X_* *D_X_*; (φ −φ φ −φ φ −φ); **k**_11_τ_9_(*M*_5_^+^); *V_B_* *D_X_*; (φ φ φ φ φ φ); **k**_11_τ_10_(*M*_5_^−^); *O_X_* *D_A_* *D_B_* *D_X_* σ; **k**_12_τ_4_(Γ_2_^−^); (σ σ σ); **k**_12_τ_7_(Γ_5_^+^) Ψ; **k**_13_τ_2_(*R*_1_^−^); Ψ; **k**_13_τ_3_(*R*_2_^+^); (Ψ Ψ Ψ); **k**_13_τ_8_(*R*_5_^−^); *V_B_* *D_X_*; (Ψ Ψ Ψ); **k**_13_τ_9_(*R*_4_^+^)	*R*3*c* (No. 161)	2**a**_1_−2**a**_2_, 2**a**_2_−2**a**_3_, 2**a**_1_+2**a**_2_+2**a**_3_ (0,0,0)	8	*A* ^6*a*^_1/4_ *A* ^18*b*^_3/4_ *B* ^6*a*^_1/4_ *B* ^18*b*^_3/4_ *X* ^18*b*(4)^_3_
15	(φ φ φ φ φ φ); **k**_11_τ_10_(*M*_5_^−^); *O_X_* *D_A_* *D_B_* *D_X_* or (σ σ σ); **k**_11_τ_10_(Γ_4_^−^); *P* (φ −φ φ −φ φ −φ); **k**_11_τ_9_(*M*_5_^+^); *V_B_* *D_X_* or (σ σ σ); **k**_11_τ_10_(Γ_4_^−^); *P* (φ φ φ); **k**_11_τ_1_(*M*_1_^+^); *O_A_* *D_X_* or (σ σ σ); **k**_11_τ_10_(Γ_4_^−^); *P* (φ φ φ); **k**_11_τ_7_(*M*_4_^+^); *O_B_O_X_D_X_*	(φ φ φ); **k**_11_τ_1_(*M*_1_^+^); *O_A_* *D_X_*; (φ φ φ); **k**_11_τ_4_(*M*_3_^−^); *D_A_*; (φ φ φ); **k**_11_τ_6_(*M*_2_^−^); *D_B_* *D_X_*; (φ φ φ); **k**_11_τ_7_(*M*_4_^+^); *O_B_* *O_X_* *D_X_*; (φ −φ φ −φ φ −φ); **k**_11_τ_9_(*M*_5_^+^); *V_B_* *D_X_*; (φ φ φ φ φ φ); **k**_11_τ_10_(*M*_5_^−^); *O_X_* *D_A_* *D_B_* *D_X_*; σ; **k**_12_τ_4_(Γ_2_^−^); (σ σ σ); **k**_12_τ_7_(Γ_5_^+^); (σ σ σ); **k**_11_τ_10_(Γ_4_^−^); *P*	*R*3*m* (No. 160)	2**a**_1_−2**a**_3_, −2**a**_2_+2**a**_3_, −**a**_1_−**a**_2_−**a**_3_ (0,0,0)	4	*A* ^3*a*^_1/4_ *A* ^9*b*^_3/4_ *B* ^3*a*^_1/4_ *B* ^9*b*^_3/4_ *X* ^9*b*(2)^_6/4_ *X* ^18*c*^_6/4_

## Footnotes

1 In this article, we consider the ideal perovskite structure as an archetype, and ideal cation and anion superstructures of perovskites as aristotypes [see details on this issue in the work of Talanov *et al.* (2016 ▸)]. By definition, group–subgroup relations necessarily exist between the space groups of the parent structure (archetype and aristotype) and the observed one. This can then be qualified as distorted (and/or ordered) structures and can be described as the parent crystalline structure plus a static symmetry-breaking structural distortion (and/or ordering).

2 The correspondence of designations by Kovalev (1993 ▸) and Miller & Love (1967 ▸) is as follows. For **k**_10_(*X*): τ_1_(*X*_1_^+^), τ_2_(*X*_1_^−^), τ_3_(*X*_3_^+^), τ_4_(*X*_3_^−^), τ_5_(*X*_2_^+^), τ_6_(*X*_2_^−^), τ_7_(*X*_4_^+^), τ_8_(*X*_4_^−^), τ_9_(*X*_5_^+^), τ_10_(*X*_5_^−^). For **k**_11_(*M*): τ_1_(*M*_1_^+^), τ_2_(*M*_1_^−^), τ_3_(*M*_3_^+^), τ_4_(M_3_^−^), τ_5_(*M*_2_^+^), τ_6_(*M*_2_^−^), τ_7_(*M*_4_^+^), τ_8_(*M*_4_^−^), τ_9_(*M*_5_^+^), τ_10_(*M*_5_^−^). For **k**_12_(Γ): τ_1_(Γ_1_^+^), τ_2_(Γ_1_^−^), τ_3_(Γ_2_^+^), τ_4_(Γ_2_^−^), τ_5_(Γ_3_^+^), τ_6_(Γ_3_^−^), τ_7_(Γ_5_^+^), τ_8_(Γ_5_^−^), τ_9_(Γ_4_^+^), τ_10_(Γ_4_^−^). For **k**_13_(*R*): τ_1_(*R*_1_^+^), τ_2_(*R*_1_^−^), τ_3_(*R*_2_^+^), τ_4_(*R*_2_^–^), τ_5_(*R*_3_^+^), τ_6_(*R*_3_^−^), τ_7_(*R*_5_^+^), τ_8_(*R*_5_^−^), τ_9_(*R*_4_^+^), τ_10_(*R*_4_^−^).

3 A set of space-group irreps of the parent cubic perovskite phase, basic functions of which make it possible to determine the atom displacements (including non-polar and polar displacements) at 1*a*, 1*b* and 3*c* Wyckoff positions of the  space group, is a mechanical (or atom-displacement) representation of the perovskite structure.

4 A set of space-group irreps of the parent cubic perovskite phase, basic functions of which make it possible to determine the atom permutations at 1*a*, 1*b* and 3*c* Wyckoff positions of the  space group, is a permutational representation of the perovskite structure.

5 From the aristotype under consideration, the structure of these perovskites is distinguished by the tilts of anion octahedra. The structural mechanisms of the formation of 1322 perovskites will be discussed in detail in Section 3.2.2.

## References

[bb1] Adams, T. B., Sinclair, D. C. & West, A. R. (2006). *Phys. Rev. B*, **73**, 094124.

[bb2] Akizuki, Y., Yamada, I., Fujita, K., Nishiyama, N., Irifune, T., Yajima, T., Kageyama, H. & Tanaka, K. (2013). *Inorg. Chem.* **52**, 11538–11543. 10.1021/ic401855j 24028492

[bb3] Akizuki, Y., Yamada, I., Fujita, K., Taga, K., Kawakami, T., Mizumaki, M. & Tanaka, K. (2015). *Angew. Chem. Int. Ed.* **54**, 10870–10874. 10.1002/anie.201504784 26211745

[bb4] Aleksandrov, K. S. (1976). *Ferroelectrics*, **14**, 801–805.

[bb5] Aleksandrov, K. S. (1978). *Ferroelectrics*, **20**, 61–67.

[bb6] Aleksandrov, K. S., Anistratov, A. T., Beznosikov, B. V. & Fedoseeva, N. V. (1981). *Phase Transitions in Crystal Halides ABX_3_*. Novosibirsk: Nauka.

[bb7] Aleksandrov, K. S. & Bartolomé, J. (2001). *Phase Transit.* **74**, 255–335.

[bb8] Aleksandrov, K. S. & Beznosikov, B. V. (2007). *Crystallogr. Rep.* **52**, 28–36.

[bb9] Aleksandrov, K. S. & Beznosikov, V. V. (1997). *Phys. Solid State*, **39**, 695–715.

[bb10] Aroyo, M. I., Kirov, A., Capillas, C., Perez-Mato, J. M. & Wondratschek, H. (2006). *Acta Cryst.* A**62**, 115–128. 10.1107/S0108767305040286 16489249

[bb11] Aso, R., Kan, D., Shimakawa, Y. & Kurata, H. (2014). *Cryst. Growth Des.* **14**, 2128–2132.

[bb12] Avdeev, M. & Nalbandyan, V. B. (2006). *Inorg. Chem.* **45**, 2217–2220. 10.1021/ic051413+ 16499386

[bb13] Balachandran, P. V. & Rondinelli, J. M. (2013). *Phys. Rev. B*, **88**, 054101.

[bb14] Bärnighausen, H. (1975). *Acta Cryst.* A**31**, S3.

[bb15] Bärnighausen, H. (1980). *Commun. Math. Chem.* **9**, 139–175.

[bb16] Belik, A. A. (2018). *Dalton Trans.* **47**, 3209–3217. 10.1039/c7dt04490a 29384532

[bb17] Belik, A. A., Glazkova, Y. S., Katsuya, Y., Tanaka, M., Sobolev, A. V. & Presniakov, I. A. (2016). *Phys. Chem. C*, **120**, 8278–8288.

[bb18] Belik, A. A., Glazkova, Y. S., Terada, N., Matsushita, Y., Sobolev, A. V., Presniakov, I. A., Tsujii, N., Nimori, S., Takehana, K. & Imanaka, Y. (2016). *Inorg. Chem.* **55**, 6169–6177. 10.1021/acs.inorgchem.6b00774 27229299

[bb19] Belik, A. A., Matsushita, Y. & Khalyavin, D. D. (2017). *Angew. Chem. Int. Ed.* **56**, 10423–10427. 10.1002/anie.201704798 28670864

[bb20] Belik, A. A., Matsushita, Y., Kumagai, Yu., Katsuya, Y., Tanaka, M., Stefanovich, S. Yu., Lazoryak, B. I., Oba, F. & Yamaura, K. (2017). *Inorg. Chem.* **56**, 12272–12281. 10.1021/acs.inorgchem.7b01723 28949543

[bb21] Birman, J. L. (1978). *Group-Theoretical Methods in Physics*, edited by P. Kramers & A. Reickers, pp. 203–222. Berlin: Springer Verlag.

[bb22] Bochu, B., Buevoz, J. L., Chenavas, J., Collomb, A., Joubert, J. C. & Marezio, M. (1980). *Solid State Commun.* **36**, 133–138.

[bb23] Bochu, B., Chenavas, J., Joubert, J. C. & Marezio, M. (1974). *J. Solid State Chem.* **11**, 88–93.

[bb24] Bochu, B., Deschizeaux, M. N., Joubert, J. C., Collomb, A., Chenavas, J. & Marezio, M. (1979). *J. Solid State Chem.* **29**, 291–298.

[bb25] Bock, O. & Müller, U. (2002). *Acta Cryst.* B**58**, 594–606. 10.1107/s0108768102001490 12149548

[bb26] Boström, H. L. B., Senn, M. S. & Goodwin, A. L. (2018). *Nat. Commun.* **9**, 2380. 10.1038/s41467-018-04764-x PMC6006342 29915202

[bb27] Bruce, A. D. & Cowley, R. A. (1981). *Structural Phase Transitions.* London: Taylor and Francis.

[bb28] Byeon, S.-H., Lee, S.-S., Parise, J. B., Woodward, P. M. & Hur, N. H. (2005). *Chem. Mater.* **17**, 3552–3557.

[bb29] Byeon, S.-H., Lufaso, M. W., Parise, J. B., Woodward, P. M. & Hansen, T. (2003). *Chem. Mater.* **15**, 3798–3804.

[bb30] Campbell, B., Howard, C. J., Averett, T. B., Whittle, T. A., Schmid, S., Machlus, S., Yost, C. & Stokes, H. T. (2018). *Acta Cryst.* A**74**, 408–424. 10.1107/S2053273318009713 30182930

[bb31] Campbell, B. J., Stokes, H. T., Tanner, D. E. & Hatch, D. M. (2006). *J. Appl. Cryst.* **39**, 607–614.

[bb32] Carpenter, M. A. & Howard, C. J. (2009). *Acta Cryst.* B**65**, 134–146. 10.1107/S0108768109000974 19299870

[bb33] Chechin, G. M. (1989). *Comput. Math. Appl.* **17**, 255–278.

[bb34] Chen, W., Mizumaki, M., Saito, T. & Shimakawa, Y. (2013). *Dalton Trans.* **42**, 10116–10120. 10.1039/c3dt50489a 23567543

[bb35] Chen, W., Mizumaki, M., Seki, H., Senn, M. S., Saito, T., Kan, D., Attfield, J. P. & Shimakawa, Y. (2014). *Nat. Commun.* **5**, 3909. 10.1038/ncomms4909 24849185

[bb36] Chen, W.-T., Long, Y., Saito, T., Attfield, J. P. & Shimakawa, Y. (2010). *J. Mater. Chem.* **20**, 7282–7286.

[bb37] Chenavas, J., Sayetat, F., Collomb, A., Joubert, J. C. & Marezio, M. (1975). *Solid State Commun.* **16**, 1129–1132.

[bb38] Cohen, M. H., Neaton, J. B., He, L. & Vanderbilt, D. (2003). *J. Appl. Phys.* **94**, 3299–3306.

[bb39] Deng, H., Liu, M., Dai, J., Hu, Z., Kuo, C., Yin, Y., Yang, J., Wang, X., Zhao, Q., Xu, Y., Fu, Z., Cai, J., Guo, H., Jin, K., Pi, T., Soo, Y., Zhou, G., Cheng, J., Chen, K., Ohresser, P., Yang, Y. F., Jin, C., Tjeng, L. H. & Long, Y. (2016). *Phys. Rev. B*, **94**, 024414.

[bb40] Deschanvres, A., Raveau, B. & Tollemer, F. (1967). *Bull. Chim. Soc. Fr.* pp. 4077–4078.

[bb41] Dimmock, J. (1963). *Phys. Rev.* **130**, 1337–1344.

[bb42] Duyker, S. G., Hill, J. A., Howard, C. J. & Goodwin, A. L. (2016). *J. Am. Chem. Soc.* **138**, 11121–11123. 10.1021/jacs.6b06785 27533044

[bb43] Ebbinghaus, S. G. (2007). *Prog. Solid State Chem.* **35**, 421–431.

[bb44] Fedorov, E. S. (1891). *Simmetriia Pracil’nykh Sistem Figur* *Zap. Min. Obshch.* **XXXVIII**, 1.

[bb45] Giovanoli, D. & Leuenberger, U. (1969). *Helv. Chim. Acta*, **52**, 2333–2347.

[bb46] Glazer, A. M. (1972). *Acta Cryst.* B**28**, 3384–3392.

[bb47] Glazer, A. M. (1975). *Acta Cryst.* A**31**, 756–762.

[bb48] Glazkova, Y. S., Terada, N., Matsushita, Y., Katsuya, Y., Tanaka, M., Sobolev, A. V., Presniakov, I. A. & Belik, A. A. (2015). *Inorg. Chem.* **54**, 9081–9091. 10.1021/acs.inorgchem.5b01472 26322969

[bb49] Goldschmidt, V. M. (1926). *Naturwissenschaften*, **14**, 477–485.

[bb50] Guo, H., Fernández-Díaz, M. T., Zhou, L., Yin, Y., Long, Y. & Komarek, A. C. (2017). *Sci. Rep.* **7**, 45939. 10.1038/srep45939 PMC5381003 28378833

[bb52] Hatch, D. M. & Stokes, H. T. (2002). *Phys. Rev. B*, **65**, 114113.

[bb53] Hermann, C. (1928). *Z. Kristallogr.* **68**, 257–287.

[bb54] Homes, C. C., Vogt, T., Shapiro, S. M., Wakimoto, S. & Ramirez, A. P. (2001). *Science*, **293**, 673–676. 10.1126/science.1061655 11474105

[bb55] Howard, C. J. & Carpenter, M. A. (2010). *Acta Cryst.* B**66**, 40–50. 10.1107/S0108768109048010 20101082

[bb56] Howard, C. J., Kennedy, B. J. & Woodward, P. M. (2003). *Acta Cryst.* B**59**, 463–471. 10.1107/s0108768103010073 12947230

[bb57] Howard, C. J. & Stokes, H. T. (1998). *Acta Cryst.* B**54**, 782–789.

[bb58] Howard, C. J. & Stokes, H. T. (2004). *Acta Cryst.* B**60**, 674–684. 10.1107/S0108768104019901 15534377

[bb59] Howard, C. J. & Stokes, H. T. (2005). *Acta Cryst.* A**61**, 93–111.

[bb60] Imamura, N., Karppinen, M., Motohashi, T., Fu, D., Itoh, M. & Yamauchi, H. (2008). *J. Am. Chem. Soc.* **130**, 14948–14949. 10.1021/ja806487d 18928263

[bb61] *International Tables for Crystallography* (2010). Vol. A1, *Symmetry Relations between Space Groups*, edited by H. Wondratschek & U. Müller, 2nd ed. Chichester: John Wiley and Sons.

[bb62] Jeitschko, W. & Braun, D. (1977). *Acta Cryst.* B**33**, 3401–3406.

[bb63] Johnson, R. D., Chapon, L. C., Khalyavin, D. D., Manuel, P., Radaelli, P. G. & Martin, C. (2012). *Phys. Rev. Lett.* **108**, 067201. 10.1103/PhysRevLett.108.067201 22401114

[bb64] Johnson, R. D., Khalyavin, D. D., Manuel, P., Radaelli, P. G., Glazkova, I. S., Terada, N. & Belik, A. A. (2017). *Phys. Rev. B*, **96**, 054448.

[bb65] Keppens, V., Mandrus, D., Sales, B. C., Chakoumakos, B. C., Dai, P., Coldea, R., Maple, M. B., Gajewski, D. A., Freeman, E. J. & Bennington, S. (1998). *Nature*, **395**, 876–878.

[bb66] Kida, T., Kammuri, R., Hagiwara, M., Yoshii, S., Kobayashi, W., Iwakawa, M. & Terasaki, I. (2012). *Phys. Rev. B*, **85**, 195122.

[bb67] King, G. & Woodward, P. M. (2010). *J. Mater. Chem.* **20**, 5785–5796.

[bb68] Kobayashi, W., Terasaki, I., Takeya, J., Tsukada, I. & Ando, Y. (2004). *J. Phys. Soc. Jpn*, **73**, 2373–2376.

[bb69] Kovalev, O. V. (1993). *Representations of Crystallographic Space Groups. Irreducible Representations, Induced Representations and Co-representations.* London: Taylor and Francis.

[bb70] Landau, L. D. (1937). *Zh. Eksp. Teor. Fiz.* **7**, 19–40.

[bb71] Landau, L. D. & Lifshitz, E. M. (1976). *Course of Theoretical Physics*, Vol. 5, *Statistical Physics.* Moscow: Nauka (Oxford: Pergamon, 1980).

[bb72] Leinenweber, K., Linton, J., Navrotsky, A., Fei, Y. & Parise, J. B. (1995). *Phys. Chem. Miner.* **22**, 251–258.

[bb73] Li, J., Subramanian, M. A., Rosenfeld, H. D., Jones, C. Y., Toby, B. H. & Sleight, A. W. (2004). *Chem. Mater.* **16**, 5223–5225.

[bb74] Lifshitz, E. M. (1941). *Zh. Eksp. Teor. Fiz.* **11**, 255–269.

[bb75] Liu, Y., Withers, R. L. & Wei, X. Y. (2005). *Phys. Rev. B*, **72**, 134104.

[bb76] Long, Y. (2016). *Chin. Phys. B*, **25**, 078108.

[bb77] Long, Y., Saito, T., Tohyama, T., Oka, K., Azuma, M. & Shimakawa, Y. (2009). *Inorg. Chem.* **48**, 8489–8492. 10.1021/ic901128k 19655792

[bb78] Long, Y. & Shimakawa, Y. (2010). *New J. Phys.* **12**, 063029.

[bb79] Long, Y. W., Hayashi, N., Saito, T., Azuma, M., Muranaka, S. & Shimakawa, Y. (2009). *Nature*, **458**, 60–63. 10.1038/nature07816 19262669

[bb80] Lotgering, F. K. (1959). *J. Inorg. Nucl. Chem.* **9**, 113–123.

[bb81] Megaw, H. D. (1957). *Ferroelectricity in Crystals.* London: Methuen.

[bb82] Megaw, H. D. (1973). *Crystal Structures – a Working Approach.* Philadelphia: W. B. Saunders.

[bb83] Megaw, H. D. & Darlington, C. N. W. (1975). *Acta Cryst.* A**31**, 161–173.

[bb84] Meng, J., Zhang, L., Yao, F., Zhang, X., Zhang, W., Liu, X., Meng, J. & Zhang, H. (2017). *Inorg. Chem.* **56**, 6371–6379. 10.1021/acs.inorgchem.7b00458 28489392

[bb85] Mezzadri, F., Buzzi, M., Pernechele, C., Calestani, G., Solzi, M., Migliori, A. & Gilioli, E. (2011). *Chem. Mater.* **23**, 3628–3635.

[bb86] Mezzadri, F., Calestani, G., Calicchio, M., Gilioli, E., Bolzoni, F., Cabassi, R., Marezio, M. & Migliori, A. (2009). *Phys. Rev. B*, **79**, 100106.

[bb87] Miller, S. C. & Love, W. F. (1967). *Tables of Irreducible Representations of Space Groups and Co-representations of Magnetic Space Groups.* Boulder: Pruett.

[bb88] Mitchel, R. H. (2002). *Perovskites, Modern and Ancient.* Canada: Almaz Press.

[bb89] Molokeev, M. S. & Misyul’, S. V. (2012). *Phys. Solid State*, **54**, 155–165.

[bb90] Moriyama, T., Kan, A. & Ogawa, H. (2013). *Mater. Sci. Eng. B*, **178**, 875–880.

[bb91] Mouron, P. & Choisnet, J. (1987). *J. Solid State Chem.* **66**, 311–317.

[bb92] Müller, U. (2004). *Z. Anorg. Allg. Chem.* **630**, 1519–1537.

[bb93] Müller, U. (2013). *Symmetry Relationships between Crystal Structures. Applications of Crystallographic Group Theory in Crystal Chemistry.* Oxford University Press.

[bb94] Nolas, G. S., Morelli, D. T. & Tritt, T. M. (1999). *Annu. Rev. Mater. Sci.* **29**, 89–116.

[bb95] Okamoto, H., Imamura, N., Karppinen, M., Yamauchi, H. & Fjellvåg, H. (2010). *Inorg. Chem.* **49**, 8709–8712. 10.1021/ic100499d 20799716

[bb96] Okamoto, H., Karppinen, M., Yamauchi, H. & Fjellvåg, H. (2009). *Solid State Sci.* **11**, 1211–1215.

[bb98] Ovsyannikov, S. V., Zainulin, Y. G., Kadyrova, N. I., Tyutyunnik, A. P., Semenova, A. S., Kasinathan, D., Tsirlin, A. A., Miyajima, N. & Karkin, A. E. (2013). *Inorg. Chem.* **52**, 11703–11710. 10.1021/ic400649h 24083336

[bb99] Perez-Mato, J. M., Orobengoa, D. & Aroyo, M. I. (2010). *Acta Cryst.* A**66**, 558–590. 10.1107/S0108767310016247 20720321

[bb100] Perks, N. J., Johnson, R. D., Martin, C., Chapon, L. C. & Radaelli, P. G. (2012). *Nat. Commun.* **3**, 1277. 10.1038/ncomms2294 23232407

[bb101] Prodi, A., Daoud-Aladine, A., Gozzo, F., Schmitt, B., Lebedev, O., van Tendeloo, G., Gilioli, E., Bolzoni, F., Aruga-Katori, H., Takagi, H., Marezio, M. & Gauzzi, A. (2014). *Phys. Rev. B*, **90**, 180101.

[bb102] Prodi, A., Gilioli, E., Gauzzi, A., Licci, F., Marezio, M., Bolzoni, F., Huang, Q., Santoro, A. & Lynn, J. W. (2004). *Nat. Mater.* **3**, 48–52. 10.1038/nmat1038 14704785

[bb103] Propach, V. (1977). *Z. Anorg. Allg. Chem.* **435**, 161–171.

[bb104] Przenioslo, R., Sosnowska, I., Suard, E., Hewat, A. & Fitch, A. N. (2002). *J. Phys. Condens. Matter*, **14**, 5747–5753.

[bb105] Rubel, M. H. K., Miura, A., Takei, T., Kumada, N., Mozahar Ali, M., Nagao, M., Watauchi, S., Tanaka, I., Oka, K., Azuma, M., Magome, E., Moriyoshi, C., Kuroiwa, Y. & Azharul Islam, A. K. M. (2014). *Angew. Chem.* **126**, 3673–3677. 10.1002/anie.201400607 24573781

[bb106] Rubel, M. H. K., Takei, T., Kumada, N., Ali, M. M., Miura, A., Tadanaga, K., Oka, K., Azuma, M., Yashima, M., Fujii, K., Magome, E., Moriyoshi, C., Kuroiwa, Y., Hester, J. R. & Avdeev, M. (2016). *Chem. Mater.* **28**, 459–465.

[bb107] Saito, T., Yamada, R., Ritter, C., Senn, M. S., Attfield, J. P. & Shimakawa, Y. (2014). *Inorg. Chem.* **53**, 1578–1584. 10.1021/ic402616r 24446735

[bb108] Sakai, Y., Yang, J., Yu, R., Hojo, H., Yamada, I., Miao, P., Lee, S., Torii, S., Kamiyama, T., Ležaić, M., Bihlmayer, G., Mizumaki, M., Komiyama, J., Mizokawa, T., Yamamoto, H., Nishikubo, T., Hattori, Y., Oka, K., Yin, Y., Dai, J., Li, W., Ueda, S., Aimi, A., Mori, D., Inaguma, Y., Hu, Z., Uozumi, T., Jin, C., Long, Y. & Azuma, M. (2017). *J. Am. Chem. Soc.* **139**, 4574–4581. 10.1021/jacs.7b01851 28240901

[bb109] Sakhnenko, V. P., Talanov, V. M. & Chechin, G. M. (1986). *Phys. Met. Metallogr.* **62**, 10–18.

[bb110] Sales, B. C., Mandrus, D. & Williams, R. K. (1996). *Science*, **272**, 1325–1328. 10.1126/science.272.5266.1325 8662465

[bb111] Sato, M. & Hama, Y. (1993). *J. Mater. Chem.* **3**, 233–236.

[bb112] Schoenflies, A. (1891). *Kristallsysteme und Kristallstruktur.* Leipzig.

[bb113] Senn, M. S. & Bristowe, N. C. (2018). *Acta Cryst.* A**74**, 308–321. 10.1107/S2053273318007441 PMC6038361 29978842

[bb114] Senn, M. S., Chen, W., Saito, T., García-Martín, S., Attfield, J. P. & Shimakawa, Y. (2014). *Chem. Mater.* **26**, 4832–4837.

[bb115] Shimakawa, Y. (2008). *Inorg. Chem.* **47**, 8562–8570. 10.1021/ic800696u 18821822

[bb116] Shimakawa, Y., Zhang, S., Saito, T., Lufaso, M. W. & Woodward, P. M. (2014). *Inorg. Chem.* **53**, 594–599. 10.1021/ic402740k 24328260

[bb117] Shimura, G., Niwa, K., Shirako, Y., Muto, M., Kusaba, K. & Hasegawa, M. (2016). *Solid State Commun.* **234–235**, 40–44.

[bb118] Shiraki, H., Saito, T., Yamada, T., Tsujimoto, M., Azuma, M., Kurata, H., Isoda, S., Takano, M. & Shimakawa, Y. (2007). *Phys. Rev. B*, **76**, 140403.

[bb119] Shiro, K., Yamada, I., Ikeda, N., Ohgushi, K., Mizumaki, M., Takahashi, R., Nishiyama, N., Inoue, T. & Irifune, T. (2013). *Inorg. Chem.* **52**, 1604–1609. 10.1021/ic3025155 23330609

[bb120] Shirokov, V. B. & Torgashev, V. I. (2004). *Crystallogr. Rep.* **49**, 20–28.

[bb121] Sinclair, D. C., Adams, T. B., Morrison, F. D. & West, A. R. (2002). *Appl. Phys. Lett.* **80**, 2153–2155.

[bb122] Sławiński, W. A., Okamoto, H. & Fjellvåg, H. (2017). *Acta Cryst.* B**73**, 313–320. 10.1107/S2052520617000725 28362296

[bb123] Sławiński, W., Przeniosło, R., Sosnowska, I. & Bieringer, M. (2010). *J. Phys. Condens. Matter*, **22**, 186001. 10.1088/0953-8984/22/18/186001 21393695

[bb124] Sławiński, W., Przeniosło, R., Sosnowska, I., Bieringer, M., Margiolaki, I. & Suard, E. (2009). *Acta Cryst.* B**65**, 535–542. 10.1107/S0108768109025300 19767675

[bb125] Stokes, H. T. & Campbell, B. J. (2017). *Acta Cryst.* A**73**, 4–13. 10.1107/S2053273316017629 28042798

[bb126] Stokes, H. T. & Hatch, D. M. (1988). *Isotropy Subgroups of the 230 Crystallographic Space Groups.* Singapore: World Scientific.

[bb51] Stokes, H. T. & Hatch, D. M. (2002). *J. Appl. Cryst.* **35**, 379.

[bb127] Stokes, H. T., Kisi, E. H., Hatch, D. M. & Howard, C. J. (2002). *Acta Cryst.* B**58**, 934–938. 10.1107/s0108768102015756 12456971

[bb128] Streltsov, S. V. & Khomskii, D. I. (2014). *Phys. Rev. B*, **89**, 201115.

[bb129] Subramanian, M. A., Li, D., Duan, N., Reisner, B. A. & Sleight, A. W. (2000). *J. Solid State Chem.* **151**, 323–325.

[bb130] Takata, K., Yamada, I., Azuma, M., Takano, M. & Shimakawa, Y. (2007). *Phys. Rev. B*, **76**, 024429.

[bb131] Talanov, M. V. (2018). *Cryst. Growth Des.* **18**, 3433–3440.

[bb132] Talanov, M. V., Shirokov, V. B., Avakyan, L. A., Talanov, V. M. & Borlakov, Kh. Sh. (2018). *Acta Cryst.* B**74**, 337–353. 10.1107/S2052520618007242 30141419

[bb133] Talanov, M. V., Shirokov, V. B. & Talanov, V. M. (2014). *Crystallogr. Rep.* **59**, 662–678.

[bb134] Talanov, M. V., Shirokov, V. B. & Talanov, V. M. (2016). *Acta Cryst.* A**72**, 222–235. 10.1107/S2053273315022147 26919374

[bb135] Talanov, V. M. (2007). *Glass Phys. Chem.* **33**, 620–635.

[bb136] Talanov, V. M. & Shirokov, V. B. (2014). *Acta Cryst.* A**70**, 49–63. 10.1107/S2053273313027605 24419170

[bb137] Talanov, V. M., Shirokov, V. B. & Talanov, M. V. (2015). *Acta Cryst.* A**71**, 301–318. 10.1107/S2053273315003848 25921499

[bb138] Talanov, V. M., Talanov, M. V. & Shirokov, V. B. (2014). *Crystallogr. Rep.* **59**, 650–661.

[bb139] Thomas, N. W. & Beitollahi, A. (1994). *Acta Cryst.* B**50**, 549–560.

[bb140] Tilley, R. J. D. (2016). *Perovskites*: *Structure–Property Relationships.* New York: John Wiley & Sons.

[bb141] Tohyama, T., Senn, M. S., Saito, T., Chen, W.-T., Tang, C. C., Attfield, J. P. & Shimakawa, Y. (2013). *Chem. Mater.* **25**, 178–183.

[bb142] Toledano, J. C. & Toledano, P. (1987). *The Landau Theory of Phase Transitions.* Singapore: World Scientific.

[bb143] Toledano, P. & Dmitriev, V. V. (1996). *Reconstructive Phase Transitions: in Crystals and Quasicrystals.* Singapore: World Scientific.

[bb144] Torgashev, V. I., Shirokov, V. B., Prokhorov, A. S. & Shuvalov, L. A. (2005). *Crystallogr. Rep.* **50**, 637–645.

[bb145] Toyoda, M., Saito, T., Yamauchi, K., Shimakawa, Y. & Oguchi, T. (2015). *Phys. Rev. B*, **92**, 014420.

[bb146] Troyanchuk, I. O. & Chobot, A. N. (1997). *Crystallogr. Rep.* **42**, 983–989.

[bb147] Tselev, A., Brooks, C. M., Anlage, S. M., Zheng, H., Salamanca-Riba, L., Ramesh, R. & Subramanian, M. A. (2004). *Phys. Rev. B*, **70**, 144101.

[bb148] Vasil’ev, A. N. & Volkova, O. S. (2007). *Low Temp. Phys.* **33**, 895–914.

[bb149] Vinberg, E. B., Gufan, Yu. M., Sakhnenko, V. P. & Sirotin, Y. I. (1974). *Kristallografiya*, **19**, 21–26.

[bb150] Wang, X., Chai, Y., Zhou, L., Cao, H., Cruz, C., Yang, J., Dai, J., Yin, Y., Yuan, Z., Zhang, S., Yu, R., Azuma, M., Shimakawa, Y., Zhang, H., Dong, S., Sun, Y., Jin, C. & Long, Y. (2015). *Phys. Rev. Lett.* **115**, 087601. 10.1103/PhysRevLett.115.087601 26340207

[bb151] Wei, W., Li, W., Butler, K. T., Feng, G., Howard, C. J., Carpenter, M. A., Lu, P., Walsh, A. & Cheetham, A. K. (2018). *Angew. Chem. Int. Ed.* **57**, 8932–8936. 10.1002/anie.201803176 29845741

[bb152] Woodward, P. M. (1997*a*). *Acta Cryst.* B**53**, 32–43.

[bb153] Woodward, P. M. (1997*b*). *Acta Cryst.* B**53**, 44–66.

[bb154] Wu, Y., Binford, T., Hill, J. A., Shaker, S., Wang, J. & Cheetham, A. K. (2018). *Chem. Commun.* **54**, 3751–3754. 10.1039/c8cc00907d 29589865

[bb155] Yagi, S., Yamada, I., Tsukasaki, H., Seno, A., Murakami, M., Fujii, H., Chen, H., Umezawa, N., Abe, H., Nishiyama, N. & Mori, S. (2015). *Nat. Commun.* **6**, 8249. 10.1038/ncomms9249 PMC4579779 26354832

[bb156] Yamada, I. (2017). *Sci. Technol. Adv. Mater.* **18**, 541–548. 10.1080/14686996.2017.1350557 PMC5613907 28970864

[bb157] Yamada, I., Etani, H., Tsuchida, K., Marukawa, S., Hayashi, N., Kawakami, T., Mizumaki, M., Ohgushi, K., Kusano, Y., Kim, Y., Tsuji, N., Takahashi, R., Nishiyama, N., Inoue, T., Irifune, T. & Takano, M. (2013). *Inorg. Chem.* **52**, 13751–13761. 10.1021/ic402344m 24224928

[bb158] Yamada, I., Murakami, M., Hayashi, N. & Mori, S. (2016). *Inorg. Chem.* **55**, 1715–1719. 10.1021/acs.inorgchem.5b02623 26815133

[bb159] Yamada, I., Takata, K., Hayashi, N., Shinohara, S., Azuma, M., Mori, S., Muranaka, S., Shimakawa, Y. & Takano, M. (2008). *Angew. Chem. Int. Ed.* **47**, 7032–7035. 10.1002/anie.200801482 18666298

[bb160] Yamada, I., Tsuchida, K., Ohgushi, K., Hayashi, N., Kim, J., Tsuji, N., Takahashi, R., Matsushita, M., Nishiyama, N., Inoue, T., Irifune, T., Kato, K., Takata, M. & Takano, M. (2011). *Angew. Chem. Int. Ed.* **50**, 6579–6582. 10.1002/anie.201102228 21648043

[bb161] Yao, W., Guo, Y.-Y. & Lightfoot, P. (2017). *Dalton Trans.* **46**, 13349–13351. 10.1039/c7dt03468g 28945261

[bb162] Zhang, G., Dong, S., Yan, Z., Guo, Y., Zhang, Q., Yunoki, S., Dagotto, E. & Liu, J.-M. (2011*a*). *Phys. Rev. B*, **84**, 174413.

[bb163] Zhang, G., Li, G., Huang, F., Liao, F., Li, K., Wang, Y. & Lin, J. (2011*b*). *J. Alloys Compd.* **509**, 9804–9808.

[bb164] Zhang, L., Terada, N., Johnson, R. D., Khalyavin, D. D., Manuel, P., Katsuya, Y., Tanaka, M., Matsushita, Y., Yamaura, K. & Belik, A. A. (2018). *Inorg. Chem.* **57**, 5987–5998. 10.1021/acs.inorgchem.8b00479 29722530

[bb165] Zhang, S., Saito, T., Chen, W.-T., Mizumaki, M. & Shimakawa, Y. (2013). *Inorg. Chem.* **52**, 10610–10614. 10.1021/ic401633c 23978188

[bb166] Zhang, S., Saito, T., Mizumaki, M., Chen, W.-T., Tohyama, T. & Shimakawa, Y. (2013). *J. Am. Chem. Soc.* **135**, 6056–6060. 10.1021/ja308851f 23088383

[bb167] Zhang, S., Saito, T., Mizumaki, M. & Shimakawa, Y. (2014). *Chem. Eur. J.* **20**, 9510–9513. 10.1002/chem.201403692 24975031

[bb168] Zhou, L., Dai, J., Chai, Y., Zhang, H., Dong, S., Cao, H., Calder, S., Yin, Y., Wang, X., Shen, X., Liu, Z., Saito, T., Shimakawa, Y., Hojo, H., Ikuhara, Y., Azuma, M., Hu, Z., Sun, Y., Jin, C. & Long, Y. (2017). *Adv. Mater.* **29**, 1703435. 10.1002/adma.201703435 28991383

[bb169] Zhu, Y., Zheng, J. C., Wu, L., Frenkel, A. I., Hanson, J., Northrup, P. & Ku, W. (2007). *Phys. Rev. Lett.* **99**, 037602. 10.1103/PhysRevLett.99.037602 17678327

[bb170] Zubkova, N. V., Arakcheeva, A. V., Pushcharovskii, D. Yu., Semenov, E. I. & Atencio, D. (2000). *Crystallogr. Rep.* **45**, 210–214.

